# Comprehensive Stress-Based De Novo Transcriptome Assembly and Annotation of Guar (*Cyamopsis tetragonoloba* (L.) Taub.): An Important Industrial and Forage Crop

**DOI:** 10.1155/2019/7295859

**Published:** 2019-10-08

**Authors:** Fahad Al-Qurainy, Aref Alshameri, Abdel-Rhman Gaafar, Salim Khan, Mohammad Nadeem, Abdulhafed Abdullah Alameri, Mohamed Tarroum, Muhammad Ashraf

**Affiliations:** Department of Botany and Microbiology, College of Science, King Saud University, Riyadh 11451, Saudi Arabia

## Abstract

The forage crop Guar (*Cyamopsis tetragonoloba* (L.) Taub.) has the ability to endure heat, drought, and mild salinity. A complete image on its genic architecture will promote our understanding about gene expression networks and different tolerance mechanisms at the molecular level. Therefore, whole mRNA sequence approach on the Guar plant was conducted to provide a snapshot of the mRNA information in the cell under salinity, heat, and drought stresses to be integrated with previous transcriptomic studies. RNA-Seq technology was employed to perform a 2 × 100 paired-end sequencing using an Illumina HiSeq 2500 platform for the transcriptome of leaves of *C. tetragonoloba* under normal, heat, drought, and salinity conditions. Trinity was used to achieve a *de novo* assembly followed by gene annotation, functional classification, metabolic pathway analysis, and identification of SSR markers. A total of 218.2 million paired-end raw reads (~44 Gbp) were generated. Of those, 193.5M paired-end reads of high quality were used to reconstruct a total of 161,058 transcripts (~266 Mbp) with N50 of 2552 bp and 61,508 putative genes. There were 6463 proteins having >90% full-length coverage against the Swiss-Prot database and 94% complete orthologs against Embryophyta. Approximately, 62.87% of transcripts were blasted, 50.46% mapped, and 43.50% annotated. A total of 4715 InterProScan families, 3441 domains, 74 repeats, and 490 sites were detected. Biological processes, molecular functions, and cellular components comprised 64.12%, 25.42%, and 10.4%, respectively. The transcriptome was associated with 985 enzymes and 156 KEGG pathways. A total of 27,066 SSRs were gained with an average frequency of one SSR/9.825 kb in the assembled transcripts. This resulting data will be helpful for the advanced analysis of Guar to multi-stress tolerance.

## 1. Introduction

Cluster bean or Guar (*Cyamopsis tetragonoloba* (L.) Taub.) is a widely grown summer forage crop particularly in some Asian countries where arid and semiarid conditions are prevalent. Although it is grown primarily for forage purpose, it has several other potential uses such as the pods as fresh vegetables and the whole plant as green manure [1–3]. Due to its widespread use in multifarious industries, Guar is referred to as one of the vital industrial crops worldwide [4, 5]. In addition, Guar seeds are a rich source of high-quality gum (galactomannan) reaching up to 78-82% of the endosperm, which is widely used in pharmaceutical, food, and cosmetics industries [6].

With regard to drought resistance, Guar is contemplated as a less water requiring (nonthirsty) crop [5]. In addition, it can thrive well under high temperature regimes [6]. Some studies have been conducted to mine Guar tolerance against drought and salinity in hot regions [7–9]. In a subsequent study carried out in Saudi Arabia [10], a Guar accession “BWP 5595,” has been characterized as highly tolerant to drought and moderately tolerant to salinity under high temperature regimes. Therefore, exploration of Guar genomic resources at different levels of molecular biology seems a plausible approach of improving Guar quality and productivity, so as to achieve maximal economic gain by the farmers.

Omics studies such as genomics, transcriptomics, and metabolomics play a critical role to characterize a phenotype. Advanced genomic tools and protocols developed so far during the past two decades have provided ample information on the genomes and transcriptomes of several organisms. Similarly, a rich repository of information available on the genetic resources of Guar could be effectively used to improve quality and productivity of the crop using advanced molecular biology tools. Since genome sequencing of Guar is not yet available, its genetic resources can be characterized using the transcriptomic approach. Cost-effective and easily affordable next-generation DNA sequencing is paving the way for complete transcriptome analysis [11, 12].

Recently, RNA-Seq has been employed to characterize the transcriptome of several plant species including both model and nonmodel plants, e.g., soybean (*Glycine max* (L.) Merr.) [13], maize (*Zea mays* L.) [14–16], lentil (*Lens culinaris* Medik.) [17, 18], chickpea (*Cicer arietinum* L.) [19, 20], common bean (*Phaseolus vulgaris* L.) [21], pigeon pea (*Cajanus cajan* (L.) Millsp.) [22], faba bean (*Vicia faba* L.) [23], peanut (*Arachis hypogaea* L.) [24], mung bean (*Vigna radiata* L.) [25, 26], field pea (*Pisum sativum* L.) [27–29], honey locust (*Gleditsia triacanthos* L.) [30], and Ghaf (*Prosopis cineraria* (L.) Druce) [31].

To the best of our knowledge, only two previous studies on transcriptome analysis of *C*. *tetragonoloba* have been performed using second-generation RNA sequencing technologies. For example, Tanwar et al. [32] characterized the transcriptome of leaf tissues from two Guar varieties, namely, M-83 and RGC-1066. Rawal et al. [33] generated a RNA-Seq-based transcriptome from leaf, shoot, and flower tissues of Guar. However, neither of the two transcriptomes identified the total gene content in Guar as the mRNAs discovered only related to a specific state of the cell or tissue at a certain time. In order to maximize Guar gene repository, Illumina RNA-Seq technology was carried out to create stress-based *de novo* transcriptome assembly from Guar leaves grown under heat, drought, and salinity stresses. Trinity was used to achieve a *de novo* assembly followed by gene annotation and functional classification. Our RNA-Seq analysis generated the first comprehensive *C. tetragonoloba* reference transcriptome under normal, heat, drought, and salinity conditions. The resulting data provide a large-scale transcriptomic analysis and could be a helpful resource for the development of tools for molecular breeding of this valuable grain legume species. Moreover, it could be helpful for the advanced analysis of its gene expression.

## 2. Materials and Methods

### 2.1. Plant Material

Guar accession BWP 5595 was previously characterized for its contrasting tolerance to heat, salinity, and drought in an open field experiment in the botanic garden of King Saud University, Riyadh, Saudi Arabia [10]. Seeds were sterilized with 5% sodium hypochlorite for 10 min, then washed and soaked with distilled water for 30 min. In a pot experiment, seeds were sowed in a mix of 1 : 1 : 1 peat moss, perlite, and soil with a temperature of 25°C (day) and 22°C (night) and a photoperiod of 16 h. After 35 days and in a completely randomized design (CRD), the seedlings were subjected to four treatments (i.e., salinity stress, drought stress, heat stress, and control). For salt treatment (S), a concentration gradient of NaCl solution was used to slow down the salt injury: 50 mM for the first day, 100 mM for the second day, and finally 200 mM. For drought stress (D), plants were grown under 40% of field capacity. For the experiment under heat stress (H), the temperature was adjusted at 42°C, whereas control seedlings were watered daily (C). After 21 days had passed, three biological replicates from each treatment were harvested for subsequent RNA-Seq (leaf samples were frozen with liquid nitrogen and stored at -80°C).

### 2.2. RNA Extraction, Library Construction, and Sequencing

The RNeasy® Plant Mini Kit (QIAGEN, Germany) was used to extract RNA following the manufacturer's guidelines. DNA contamination was removed using DNase I, Bovine Pancreas, >1800 U, RNase Free (Biomatik, Wilmington, Delaware, USA). The RNA integrity number (RIN) was assessed using an Agilent 2100 Bioanalyzer instrument (Agilent Technologies, Santa Clara, CA, USA). High purity and integrity samples of RNA were coded and labeled and then shipped to the Macrogen company (Macrogen Inc., Seoul, Korea, http://www.macrogen.com) for total mRNA sequencing. A TruSeq® Stranded mRNA kit (Illumina, San Diego, CA, USA) was used to construct the cDNA library. Sequencing of 2 × 100 paired-ends was performed using an Illumina HiSeq 2500 platform (Illumina, San Diego, CA, USA).

### 2.3. Read Quality Control and Adapter Removal

FASTQC V 0.11.5 software was used to check the quality of the raw sequence data [34]. Trimmomatic V 0.36 [35] was used to filter and remove the adapter sequences and trim low-quality reads with or without ambiguous sequences “N.” The Trimmomatic parameters were set as follows: PE, phred33, leading:20, trailing:20, slidingwindow:4:25, and minlen:25. The error correction software for Illumina RNA-Seq reads Rcorrector V 1.0.3 was used to correct random sequencing errors. K-mer was set at 32, *t* was set at 3, and other parameters were set at default values [36]. The output clean reads were checked in FastQC and used as input data to the RNA-Seq *de novo* assembly. All treatment replicates were concatenated together into two files (paired-end) to be used in the assembly.

### 2.4. RNA-Seq *De Novo* Assembly and Transcriptome Assessment

For RNA-Seq *de novo* assembly, Trinity ver2.4.0 [37] was used for *de novo* transcriptome assembly and downstream analyses. Different assemblies were generated using a K-mer value at 32 and default options for other parameters [38]. The quality of the assemblies was compared using the Transcriptome Contig Nx Statistic, TransRate [39], and PRINSEQ tool [40].

In order to comprehensively capture the read alignments, Bowtie 2 [41] was used to align the reads to the transcriptome, and then the number of proper pairs and improper or orphan read alignments were counted. Finally, the Integrative Genomics Viewer (IGV) [42] was used for visualizing the read support across any of the Trinity assemblies. Evaluation of the quality of a transcriptome assembly can be done by examining the number of transcripts assembled and that appeared to be full length or nearly full length. These analyses were carried out using BLAST+ against the most famous and useful protein database, namely, Swiss-Prot. To benchmark completeness of *C. tetragonoloba* transcriptome assembly, the Benchmarking Universal Single-Copy Orthologs (BUSCO) version 3.0.1 [43] was used with the default *E*-value cut-off of 1*e* − 03 against the ortholog set of Embryophyta_odb9 lineage (creation date: 2016-02-13, number of species: 30, and number of BUSCOs: 1440) from OrthoDB v9.

To detect whether the data were assembled as strand-specific or not, the level of strand specificity of the RNA-Seq data was estimated by aligning the reads back to the Trinity assembly and the distribution of RNA-Seq. First, the reads were aligned back against the Trinity assembly using Bowtie 2. Then, the distribution of strand specificity was examined by looking at the distribution of orientations for the first read of paired-end fragment reads. For clustering nucleotide sequences by removing repetitive, identical, and near-identical transcripts, the CD-HIT-EST from the CD-HIT version 4.7 (built on July 1, 2017) package was used with a sequence identity cut-off of 100% and all the rest of the parameters were set to the default values [44, 45].

### 2.5. Gene Ontology (GO) Metabolic Signaling Pathway Analysis

The professional version of Blast2GO software suite v4.1 [46–48] (https://www.blast2go.com/) was used to carry out homology searches (BLASTX and BLASTN) of unique sequences and functional annotation by Gene Ontology (GO; http://www.geneontology.org/) terms, protein sequence analysis and classification (InterPro, EBI, https://www.ebi.ac.uk/interpro/), enzyme classification codes (EC), and Kyoto Encyclopedia of Genes and Genomes [49–51] (http://www.genome.jp/kegg/). Sequences were blasted against a nonredundant (nr) protein database belonging to the National Center for Biotechnology Information (NCBI) via BLASTx-fast using the default settings. InterPro was executed in parallel to the BLAST step followed by Gene Ontology mapping and gene annotation; then, Gene Ontology was derived from BLAST, and InterPro steps were merged together. Finally, GO Slim reduction was carried out. Furthermore, Blast2GO was used to assign biological functions, cellular components, and cellular processes as well as other useful statistics to the transcripts.

### 2.6. Mining of Simple Sequence Repeat (SSR) Markers

Commonly, transcriptome data is used as a source for the simple sequence repeat (SSR) marker. Therefore, with the aim of SSR mining and documentation of SSRs in an abiotic stressed *C. tetragonoloba* transcriptome, the microsatellite identification tool Perl script (MISA) search engine [52] (http://pgrc.ipk-gatersleben.de/misa) was employed. The minimum numbers of repeats used for selecting the SSRs were six for dinucleotide repeats and five for trinucleotide, tetranucleotide, pentanucleotide, and hexanucleotide repeats. All motifs comprising continuous uninterrupted repeats were termed as perfect, and those possessing two or more classes of repeats were categorized as compound microsatellites. Moreover, the maximal number of bases interrupting 2 SSRs in a compound microsatellite was set as 100. Statistical analysis was carried out to figure out the number of SSRs with each type of motif and the length distribution of repeat units. Mononucleotide repeats were not mined due to the presence of plenty of Poly-A in such mRNA-based transcriptomes.

## 3. Results

### 3.1. Sequencing and Quality Control

To construct the transcriptome of *C. tetragonoloba*, high-quality RNAs from three replicates of four stress conditions representing heat, drought, and salinity stresses, in addition to a normal condition (control), were sequenced. In total, 218.2 million paired-end raw reads (~44 Gbp) with an average read length of 100 bp were generated from the targeted samples (Table 1). The GC content ranged between 43.98% (H3) and 45.33% (D3) with an average of 44.5%, whereas the AT content ranged from 54.67% (D3) to 56.02% (H3) with an average of 55.5%. The ratio of reads that have a Phred quality score of over 20 (Q20) ranged from 95.34% (D1) to 97.21 (H3) with an average of 96.8%, whereas Q30 ranged from 92.77% (D1) to 95.3% (H3) with an average of 94.7% indicating high-quality reads. After checking the quality of reads, the adapter sequences were removed. The low-quality reads with or without ambiguous sequences “N” were trimmed which resulted in the dropping of about 5,748,299 paired reads (2.63%) as shown in Table 2. Forward only surviving reads (14838709 pairs, 6.8%) and reverse only surviving reads (4084933 pairs, 1.87%) were also excluded in this study, although they could be used in a single-end analysis. The remaining ~193.5M paired-end reads (88.69%) were processed for correction of random sequencing errors. Among those reads, ~38.6 Mbp were corrected (Table 2). Quality control reflected extremely high-quality reads after the trimming and bases correction procedures.

### 3.2. RNA-Seq *De Novo* Assembly

**Figure 1:**
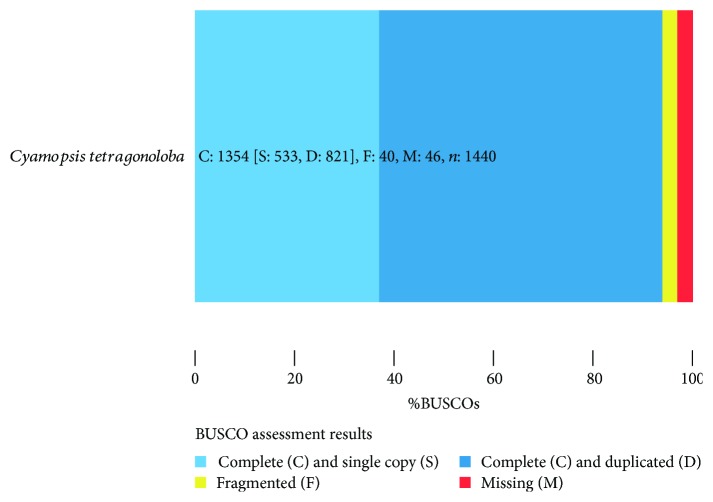
BUSCO analysis of *C. tetragonoloba* leaf transcriptome assembly completeness under normal, heat, drought, and salinity conditions.

Transcriptome construction was carried out using Trinity ver2.4.0 with a K-mer of 32 before and after the error correction made by Rcorrector (Table 3). Although use of Rcorrector software improved the quality of bases, it did not affect the quality of the whole transcriptome. Based on the results of the transcriptome made by Trinity ver2.4.0 with a K-mer of 32 after base correction using the Rcorrector software, a total of 161,058 transcripts were reconstructed into ~266 Mbp with N50 of 2552 bp and the largest transcript length of 13858 bp. A total of 95,369 transcripts had a length of more than 1 kbp, and 101 transcripts had a length of more than 10 kbp. Based on the longest isoform per gene, a total dataset of 61,508 putative genes with an average length of 1045.24 bp and an N50 of 2258 bp was obtained with an assembled size of ~64.3 Mbp. The results of the RNA-Seq read representation by Trinity assembly showed that our transcriptome assembly had the vast majority of all reads mapping back to the assembly, and 100% of the mapped fragments found mapped as proper pairs yielding concordant alignments 1 or more times to the reconstructed transcriptome. In terms of contig full-length transcripts, the distribution of percent length coverage for the top matching transcriptome entries against Swiss-Prot shows that there are 14,929 proteins that match with our transcripts. Of those, 6463 proteins (43.3%) are represented by nearly full-length transcripts, having >90% alignment coverage. Regarding completeness assessment, Figure 1 illustrates the BUSCO assessment results. Compared to the 1440 single-copy orthologs for the Embryophyta lineage, our assembly had 1354 (94%) complete BUSCOs (533 complete single-copy and 821 complete duplicated BUSCOs), while 2.8% of contigs were fragmented (40 BUSCOs) and 3.2% were missing (46 BUSCOs). Strand specificity of RNA-Seq reads was examined to determine whether the assembly of the transcriptome was made correctly. The different ratio values plotted according to top cumulative quantiles of total numbers of reads reveal obviously that the data were assembled as strand-specific which is in alignment with our used approach (dUTP approach, strand-specific library type reverse forward (--SS_lib_type RF)).

### 3.3. Gene Ontology (GO) Analysis of Transcriptome

#### 3.3.1. Blasting, Mapping, and Annotation

A total of 161,058 transcripts were subjected to analysis using Blast2GO. Out of those transcripts, 161,048 (99.99%) were with InterProScan, 101,117 (62.87%) blasted, 81,273 (50.46%) mapped, and 70,068 (43.50%) annotated (Supporting figures: [Supplementary-material supplementary-material-1]). *E*-value distribution shows that all of the 101,117 hits were at *E*‐value ≥ 1e − 4 and the most significant hits (97.27%) were at *E*‐value ≥ 1e − 180 indicating a high quality of hits and very low random background noise (Supporting figures: [Supplementary-material supplementary-material-1]). The UniProt Knowledgebase (UniProtKB) is the central hub for the collection of functional information on proteins, with accurate, consistent, and rich annotation. A significant amount of mapping data (99.23% with mapping information) was derived from the Universal Protein Resource (UniProt) database, followed by GR_protein (0.77%). As illustrated in Supporting figures: [Supplementary-material supplementary-material-1], transcript lengths ranged from 201 bp (326 transcripts) to 13855 bp (1 transcript) with an average length of 1651 bp and a total length of 265,942,016 bp. The length of 210 bp which represents 1% of transcripts recorded the highest number of transcripts (1657 transcripts).

### 3.4. Phylogenetic Analysis

**Figure 2:**
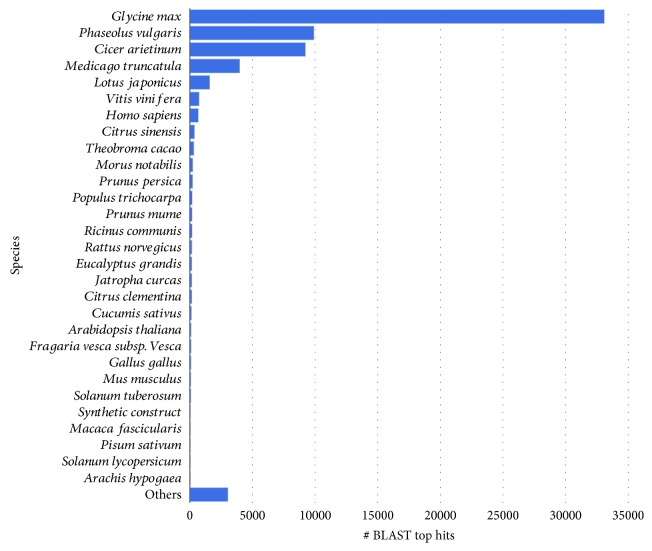
Top-hit species distribution similarity of the *C. tetragonoloba* leaf transcriptome under normal, heat, drought, and salinity conditions.

The blast results show that the transcripts examined had a top hit with 95 species (Figure 2) after removal of those of ≤10 blast hits (600 species). *Glycine max* had the highest similarity with 33,043 blast hits (50.07%), followed by *Phaseolus vulgaris* (9909; 15.02%), *Cicer arietinum* (9229; 13.98%), *Medicago truncatula* (3990; 9.05%), and *Lotus japonicus* (1601; 2.43%).

### 3.5. Protein Sequence Analysis and Classification (InterProScan, IPS)

**Figure 3:**
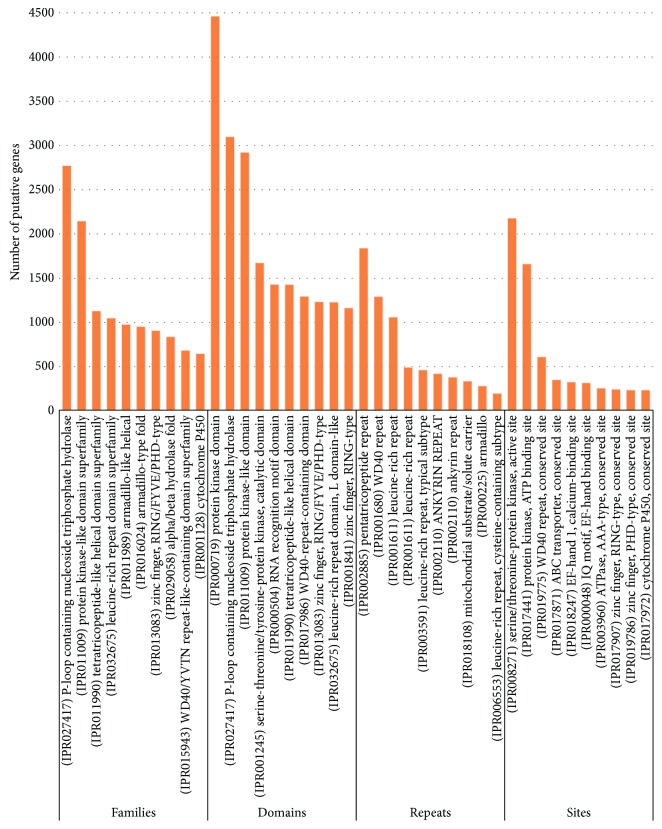
The InterProScan ID distribution (IPS features) of the *C. tetragonoloba* leaf transcriptome under normal, heat, drought, and salinity conditions.

Protein sequence analysis and classification (InterProScan, IPS) is a tool that allows sequences (protein and nucleic) to be scanned against InterPro's signatures. Out of 161,058 transcripts, there were 128,531 (79.80%) which had IPS and 69,100 (53.76%) of them had GOs. A total of 4715 IPS families were found (Figure 3).

The family (IPR027417) P-loop containing nucleoside triphosphate hydrolase had the highest number of transcripts (2765 transcripts) followed by (IPR011009) protein kinase-like domain superfamily (2139 transcripts); (IPR011990) tetratricopeptide-like helical domain superfamily (1122 transcripts); (IPR032675) leucine-rich repeat domain superfamily (1041 transcripts); (IPR011989) armadillo-like helical (969 transcripts); (IPR016024) armadillo-type fold (945 transcripts); (IPR013083) zinc finger, RING/FYVE/PHD-type (901 transcripts); (IPR029058) alpha/beta hydrolase fold (832 transcripts); (IPR015943) WD40/YVTN repeat-like-containing domain superfamily (676 transcripts); and (IPR001128) cytochrome P450 (640 transcripts).

A total of 3441 domains were detected (Figure 3). The (IPR000719) protein kinase domain matched with the highest number of transcripts (4454 transcripts), followed by (IPR027417) P-loop containing nucleoside triphosphate hydrolase (3092 transcripts); (IPR011009) protein kinase-like domain (2914 transcripts); (IPR001245) serine-threonine/tyrosine-protein kinase, catalytic domain (1665 transcripts); (IPR000504) RNA recognition motif domain (1422 transcripts); (IPR011990) tetratricopeptide-like helical domain (1421 transcripts); (IPR017986) WD40-repeat-containing domain (1287 transcripts); (IPR013083) zinc finger, RING/FYVE/PHD-type (1226 transcripts); and (IPR032675) leucine-rich repeat domain, L domain-like (1222 transcripts) as illustrated in Figure 3.

As reported in Figure 3, a total of 74 IPS repeats were detected. The (IPR002885) pentatricopeptide repeat matched with the highest number of transcripts (1832), followed by (IPR001680) WD40 repeat (1284 transcripts); (IPR001611) leucine-rich repeat (1053 transcripts); (IPR019734) tetratricopeptide repeat (485 transcripts); (IPR003591) leucine-rich repeat, typical subtype (456 transcripts); (IPR002110) ankyrin repeat (414 transcripts); and (IPR020472) G-protein beta WD40 repeat (374 transcripts).

Four hundred and ninety IPS sites were detected (Figure 3). The (IPR008271) serine/threonine-protein kinase, active site, matched with the highest number of transcripts (2171), followed by (IPR017441) protein kinase, ATP binding site (1655 transcripts); (IPR019775) WD40 repeat, conserved site (603 transcripts); (IPR017871) ABC transporter, conserved site (344 transcripts); (IPR018247) EF-hand 1, calcium-binding site (319 transcripts); (IPR000048) IQ motif, EF-hand binding site (310 transcripts); and (IPR003960) ATPase, AAA-type, conserved site (249 transcripts).

At the level of ID distribution by database, seven databases showed a match with the Guar transcriptome examined here (Supporting figures: [Supplementary-material supplementary-material-1]).

### 3.6. Functional Annotation

Of the three core GO annotation categories, biological processes (BP) comprised 64.12% of the total assigned annotations, whereas molecular functions (MF) and cellular components (CC) comprised 25.42% and 10.46%, respectively.

**Figure 4:**
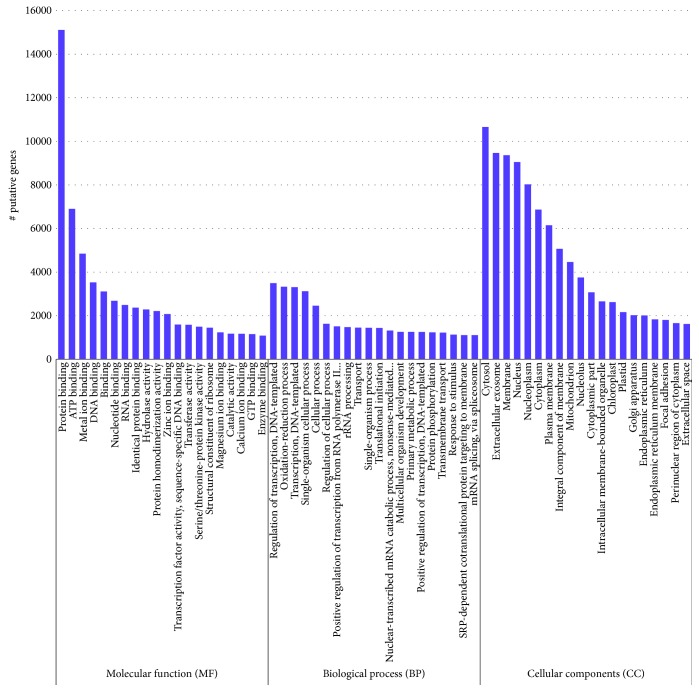
Top-20 Gene Ontology (GO) classification of the *C. tetragonoloba* transcriptome in response to normal, heat, drought, and salinity conditions.

The GO terms with the largest number of assigned transcripts in the biological process (BP) category were regulation of transcription, DNA-templated (3494; 0.97%); oxidation-reduction process (3329; 0.93%); transcription, DNA-templated (3313; 0.92%); single-organism cellular process (3120; 0.87%); cellular process (2458; 0.68%); regulation of cellular process (1630; 0.45%); and positive regulation of transcription from RNA polymerase II promoter (1510; 0.42%) (Figure 4).

Meanwhile, within the cellular component (CC) category, the terms with the most transcripts were cytosol (10662; 5.53%), extracellular exosome (9465; 4.91%), membrane (9365; 4.86%), nucleus (9054; 4.7%), nucleoplasm (8029; 4.16%), cytoplasm (6868; 3.56%), and cytosol (6151; 3.19%).

In the molecular function (MF) category, the terms with the most transcripts were protein binding (15115; 8.21%), ATP binding (6901; 3.75%), metal ion binding (4844; 2.63%), DNA binding (3529; 1.92%), nucleotide binding (2682; 1.46%), and RNA binding (2491; 1.35%).

### 3.7. KEGG Pathway Mapping

**Figure 5:**
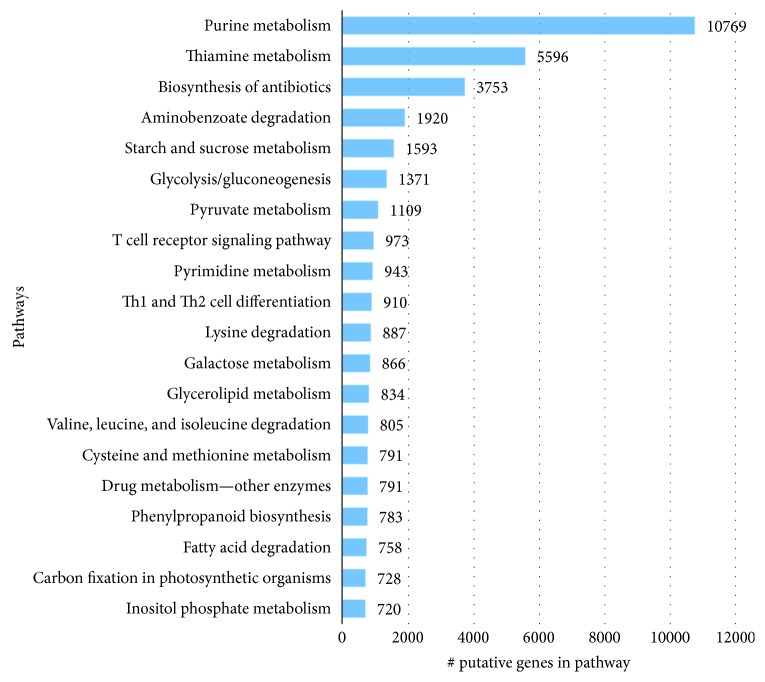
Top-20 KEGG pathways of the *C. tetragonoloba* leaf transcriptome under normal, heat, drought, and salinity conditions.

The KEGG pathway-based analysis indicated that 19,569 (12.15%) transcripts of the 161,058 transcripts obtained hits in KEGG database, and those transcripts were associated with 985 enzymes and 156 KEGG pathways (Figure 5).

**Figure 6:**
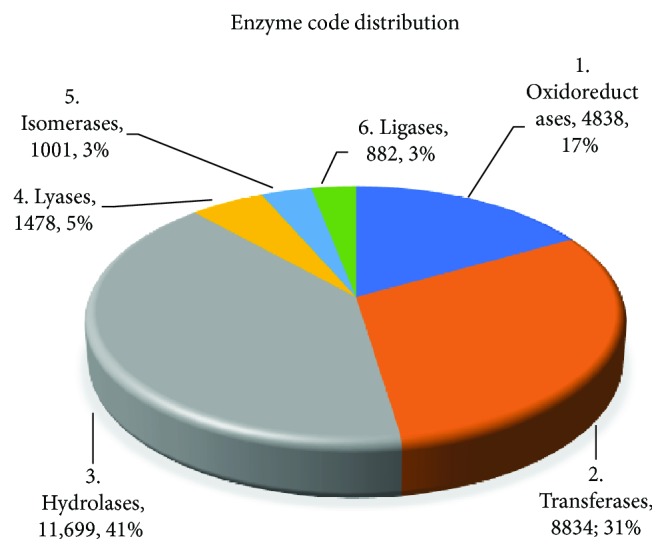
Enzyme code distribution of the *C. tetragonoloba* leaf transcriptome under normal, heat, drought, and salinity conditions.

The 985 enzymes were further categorized into 6 main classes. As demonstrated in Figure 6, hydrolases represented the highest number of transcripts (11,699; 41%), followed by transferases (8834; 31%), oxidoreductases (4838; 17%), lyases (1478; 5%), isomerases (1001; 3%), and ligases which represented the lowest number of transcripts (882; 3%). These 6 classes were recategorized to subclasses (Supporting figures: [Supplementary-material supplementary-material-1]).

### 3.8. Simple Sequence Repeat (SSR) Prognostication

**Figure 7:**
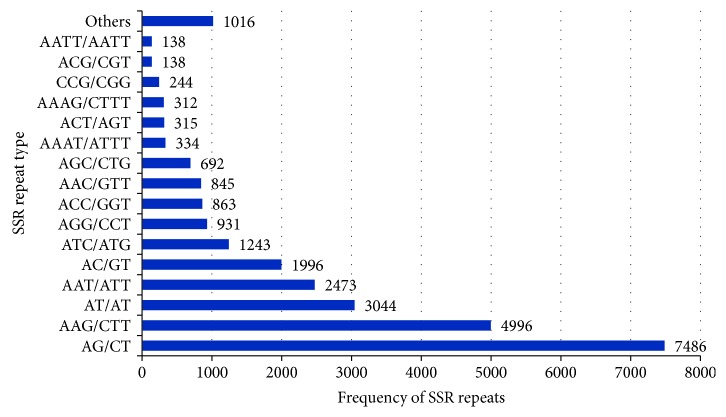
Frequency of classified repeat types of SSRs (considering sequence complementary).

From the assembled transcripts of *C. tetragonoloba* constructed under heat, drought, and salinity stress, a total of 27,066 SSRs were gained with an average frequency of one SSR per 9.825 kb in the assembled transcripts (Table 4). Out of the total 161,058 transcripts, 21,443 (13.3%) were found to contain SSR and 4289 of these transcripts had more than one SSR with 2054 of these present in compound formation. The most abundant class of repeat motifs was found to be trinucleotide (47.1%) followed by dinucleotide (46.3%) SSRs. Other repeat motifs were just a fraction of these amounting to 4.7%, 1%, and 0.8%, of tetra-, hexa-, and pentanucleotide repeats, respectively. Most of the SSRs (93.4%) were not repeated more than 10 times.  Figure 7 represents the frequency of classified repeat types considering.

## 4. Discussion

*C. tetragonoloba* (2*n* = 14) is an annual legume crop. Among the three species of the Fabaceous genus *Cyamopsis*, the recently domesticated species, *tetragonoloba*, is the only cultivated crop [53] suggesting that it is still conserving a considerable amount of its wild genetic stock which is tolerant to harsh climates. Its high tolerance for many abiotic stresses including heat, drought, and salinity is known [10] indicating that it is a highly valuable reservoir for genes that are tolerant to those abiotic stresses. To utilize this genetic tank, we implemented a *de novo* leaf transcriptome assembly of accession “BWP 5595.”

Highly restricted standards were implemented including base correction and removal of forward and reverse only surviving reads (unpaired reads) to ensure high-quality end-products. As a result, from the 218.2 million paired-end raw reads generated from the targeted samples, we could retain ~193.5 million paired-end reads of high-quality trimmed reads and corrected bases with an extremely high-quality score of >36. Although our produced data is 3.5 times higher than the previous study of the *C. tetragonoloba* leaf transcriptome [32] which produced 61.7M row paired-end reads, it is still larger than the data produced by Rawal et al. [33] who produced ~150M row paired-end reads and ~145M high-quality paired and unpaired-end reads.

To construct a highly efficient transcriptome, we used three versions of Trinity and compared the results of the constructed transcriptomes using the N50 statistical measurement. Consequently, the transcriptome that was constructed by Trinity ver2.4.0 after base correction using Rcorrector with K-mer of 32 bp has been chosen for further analysis. The generated transcriptome had a total of 161,058 transcripts (266 Mb) which is much better than Tanwar et al. [32] who generated a Guar transcriptome of 79,355 transcripts and, also, still higher than Rawal et al. [33] who generated 127,706 transcripts (179.50 Mb).

There are several yardsticks for transcriptome assembly quality assessment including N50, average length of contigs, examination of the RNA-Seq read representation of the assembly, and examination of the representation of full-length reconstructed protein-coding genes. In terms of N50 and average length of contigs, our assembly had an N50 value of 2552 bp with an average length of assembled transcript reaching 1651 bp which is better than the recently reported de novo transcriptomes among leguminous plant species. For example, chickpea (*Cicer arietinum* L.), field pea (*Pisum sativum* L.), honey locust (*Gleditsia triacanthos* L.), lentil (*Lens culinaris*), mung bean (*Vigna radiata* L.), common sainfoin (*Onobrychis viciifolia*), peanut (*Arachis hypogaea*), pigeon pea (*Cajanus cajan*), *Prosopis cineraria*, and red clover (*Trifolium pratense*) where the N50 values were in the range of 780–1930 bp and average lengths were around 520–1270 bp [17–20, 22, 24–26, 28–33, 54, 55]. In Guar (*Cyamopsis tetragonoloba*), our transcriptome had a significantly higher N50 and average length than the transcriptome of Tanwar et al. [32] who had an N50 and average length of 1035 bp and 679 bp, respectively, and slightly better than the transcriptome of Rawal et al. [33] who had an N50 and average length of 2263 bp and 1405 bp, respectively. This relative improvement in our transcriptome might be due to the use of massive high-quality reads, the latest Trinity assembler version, and maximal K-mer (32 bp).

The percentage of raw reads mapping back to the assembly is a quality metric that provides an estimation of assembly completeness [56]. Our transcriptome assembly had the vast majority of all reads mapping back to the assembly, and 100% of the mapped fragments were found mapped as proper pairs (yielding concordant alignments 1 or more times to the reconstructed transcriptome). Furthermore, our findings are superior to Parmakelis et al. [57], Diray-Arce et al. [58], and Khudyakov et al. [59] who found that 65.58%, 72.91%, and 86.60% of the total raw reads could be mapped back to the assembly. This high concordance in our transcriptome might be due to the K-mer which was set at the maximal value of 32-mer [60].

One of the most important means for appraising the quality of a transcriptome assembly is to line up the assembled transcripts against all known proteins and verify the number of unique top matching proteins that align across more than *X*% of its length. By using the Swiss-Prot sequence database [61] in our transcriptome, there are 6463 (43.3%) proteins that are covered by more than 90% of their protein lengths and 10,781 (72.2%) covered by >50%. These results are analogous to those of Chen et al. [62] who reported 7472 (39.7%) proteins.

For genome completeness assessment, both CEGMA [63] and BUSCO [43] use a similar approach: searching for a list of predefined conserved orthologous genes assumed to be present in all completed transcriptome assemblies for members of a specific clade. While CEGMA and BUSCO can be used for genome quality assessment, only BUSCO can be used for transcriptomes and proteomes [64, 65]. CEGMA is no longer being supported since May 2015; however, the new tool “BUSCO” has been published by Simão et al. [43] in the same year [66]. Using the BUSCO tool against the Embryophyta lineage which covers 30 species and 1440 BUSCOs, our transcriptome assembly had a much higher percentage of complete BUSCOs (94%). Furthermore, it has low fragmented and missed BUSCO orthologs of 2.8% and 3.2%, respectively, indicating that our transcriptome is a quality assembly with a high degree of completeness. Although CEGMA is not designed for transcriptome assessment and is even no longer being supported since 2015, both Tanwar et al. [32] and Rawal et al. [33] used the CEGMA tool to assess the *C. tetragonoloba* transcriptome and reported a completeness of 87.50% and 98.79%, respectively.

Of the 161058 transcripts of the *C. tetragonoloba* transcriptome, 161,048 (99.99%) were with InterProScan, 101,117 (62.87%) were blasted, 81,273 (50.46%) were mapped, and 70,068 (43.50%) were annotated. More than 50% of the transcripts had no significant matches. This low ratio of matching could be due to either the high cut-off, shorter transcript length, representation of the novel or Guar lineage specific transcripts, or lacking a known conserved functional domain or noncoding RNAs [32, 33, 67, 68]. In this study, we are reporting a heat-, drought-, and salinity-stressed transcriptome, and those transcripts of no hits could be of prodigious interest for further research such as identification of differentially expressed genes and single nucleotide polymorphisms (SNPs) of this valuable crop under harsh abiotic stresses.

Phylogenetically, our results showed that Guar transcripts had a top-hit similarity of >90% of leguminous species indicating a respectable coverage of the homologous legume sequence conservation with our assembled transcriptome [33, 55]. The *Glycine max* had the highest similarity (50.07%), followed by *Phaseolus vulgaris* (15.02%), *Cicer arietinum* (13.98%), *Medicago truncatula* (9.05%), and *Lotus japonicus* (2.43%). This highest similarity with *Glycine max* is in harmony with those of Tanwar et al. [32] and Rawal et al. [33] who also found a highest similarity of 41% and 31% with *G. max*, respectively, and could be due to the paucity of genomic and transcriptomic studies of the other leguminous species. Consequently, the genome of *G. max* may serve as a reference for *C. tetragonoloba* in the future studies [32].

Comparatively, transcriptome-based SSR molecular markers become more favorable and helpful due to their high cross-species transferability, high amplification rate, and being relatively inexpensive compared with the SSR markers of nontranscribed regions [69, 70]. Moreover, since they can easily reveal variation in the expressed portion of the genome, so it is possible to appraise marker-trait association and specific genomic regions expressing important physioagronomic traits [71].

In our transcriptome, a total of 27,066 prospective SSRs with a frequency of one SSR per 9.825 kb were recognized which is similar to what has been observed by Rawal et al. [33] who reported one SSR per 10.20 kb in the transcriptome of *C. tetragonoloba*. However, Kumar et al. [72] reported one SSR per 7.9 kb in EST-SSR and Rawal et al. [33] and Tanwar et al. [32] identified one SSR per 8.75 kb and one SSR per 7.31 kb in *C. tetragonoloba* unigenes, respectively, which are still not so far from our findings. Comparatively, frequency of SSRs in other legumes was one SSR per 8.4 kb, 5.80 kb, 2.94 kb, and 4.7 kb in pigeon pea, chickpea leaves and flowers, chickpea seeds, and common bean, respectively [19, 20, 73, 74], indicating that the SSR frequency in *C. tetragonoloba* is lower than that of some legumes and in the same trend with others. The differences in the overall frequency might be due to the use of different tools and criteria to identify SSRs and the size of the assembly dataset [75]. Di- and trinucleotide SSRs represented a large attribution (93.4%) which is consistent with the later study of *C. tetragonoloba* [33].

## 5. Conclusion

In our current study, RNA-Seq technology was utilized to perform sequencing of the leaves of *C. tetragonoloba* accession “BWP 5595” under normal, heat, drought, and salinity conditions. A total of 193.5M high-quality paired-end reads were employed to reconstruct a total of 161,058 transcripts and 61,508 putative genes. There are 6463 proteins having >90% full-length coverage against the Swiss-Prot database and 94% complete orthologs against Embryophyta indicating a high-quality transcriptome. In this study, our RNA-Seq analysis generated the first comprehensive abiotic stress-induced reference transcriptome for *C. tetragonoloba* under normal, heat, drought, and salinity conditions. The transcriptome data presented here will be helpful for advanced analysis of gene expression.

## Figures and Tables

**Table 1:** Raw data statistics and quality assessment.

Treatment/replicate	Total read bases (bp)	Total reads (pairs)	GC (%)	AT (%)	Q20 (%)	Q30 (%)
C1	3,682,737,548	18,231,374	44.77	55.23	97.08	95.08
C2	3,746,599,646	18,547,523	44.55	55.45	96.76	94.58
C3	3,881,717,446	19,216,423	44.49	55.51	97.0	94.98
D1	3,218,536,498	15933349	44.13	55.87	95.34	92.77
D2	3,711,544,566	18,373,983	44.8	55.2	96.97	94.93
D3	3,892,264,068	19,268,634	45.33	54.67	96.82	94.66
S1	3,775,903,180	18,692,590	44.58	55.42	97.06	95.04
S2	3,628,098,568	17,960,884	44.73	55.27	96.9	94.79
S3	3,481,866,526	17,236,963	44.32	55.68	96.9	94.82
H1	3,589,147,716	17,768,058	44.2	55.8	96.98	94.97
H2	3,541,905,774	17,534,187	44.11	55.89	96.92	94.93
H3	3,922,224,910	19,416,955	43.98	56.02	97.21	95.3
Total	44,072,546,446	218,180,923				

Total read bases: total number of bases sequenced; total reads: total number of reads. In Illumina paired-end sequencing, read1 and read2 are added. GC (%): GC content; AT (%): AT content; Q20 (%): ratio of reads having a Phred quality score of over 20; Q30 (%): ratio of reads having a Phred quality score of over 30; C: control; D: drought stress; S: salinity stress; H: heat stress; numbers 1, 2, & 3 beside the treatment name: indicates biological replicate number.

**Table 2:** Results of the trimming of adapters and low-quality reads with Trimmomatic and corrected bases with Rcorrector for each library sequenced.

Treatment/replicate	Input read (pairs)	Both surviving	Forward only surviving	Reverse only surviving	Dropped	Corrected bases
C1	18,231,374	16,315,223 (89.49%)	1,104,286 (6.06%)	343,131 (1.88%)	468,734 (2.57%)	3,367,429
C2	18,547,523	16,383,381 (88.33%)	1,312,083 (7.07%)	329,272 (1.78%)	522,787 (2.82%)	3,308,267
C3	19,216,423	17,119,827 (89.09%)	1,243,179 (6.47%)	342,799 (1.78%)	510,618 (2.66%)	3,422,805
D1	15,933,349	13,404,773 (84.13%)	1,716,786 (10.77%)	360,810 (2.26%)	450,980 (2.83%)	2,449,953
D2	18,373,983	16,366,657 (89.08%)	1,198,202 (6.52%)	328,085 (1.79%)	481,039 (2.62%)	3,222,892
D3	19,268,634	17,026,208 (88.36%)	1,387,280 (7.20%)	311,715 (1.62%)	543,431 (2.82%)	3,485,911
H1	17,768,058	15,809,295 (88.98%)	1,175,640 (6.62%)	333,378 (1.88%)	449,745 (2.53%)	3,259,472
H2	17,534,187	15,606,157 (89.00%)	1,141,252 (6.51%)	362,422 (2.07%)	424,356 (2.42%)	3,069,003
H3	19,416,955	17,451,837 (89.88%)	1,106,763 (5.70%)	390,096 (2.01%)	468,259 (2.41%)	3,538,389
S1	18,692,590	16,712,479 (89.41%)	1,140,121 (6.10%)	353,534 (1.89%)	486,456 (2.60%)	3,293,908
S2	17,960,884	15,972,952 (88.93%)	1,192,753 (6.64%)	307,947 (1.71%)	487,232 (2.71%)	3,072,708
S3	17,236,963	15,340,193 (89.00%)	1,120,364 (6.50%)	321,744 (1.87%)	454,662 (2.64%)	3,068,499
Total	218,180,923	193,508,982 (88.69%)	14,838,709 (6.80%)	4,084,933 (1.87%)	5,748,299 (2.63%)	38,559,236

C: control; D: drought stress; S: salinity stress; H: heat stress; numbers 1, 2, & 3 beside the treatment name: indicates biological replicate number.

**Table 3:** Trinity assembly evaluation (K‐mer = 32 with Rcorrector) and TransRate.

Evaluation parameters	
Counts of genes and transcripts	
Total Trinity genes	61,508
Total Trinity transcripts	161,058
GC percent	39.27
Stats based on all transcript contigs	
Contig N50	2552
Median contig length	1351
Mean contig length	1651.22
Shortest contig	201
Longest contig	13,858
Mean orf percent	44.79
Total assembled bases	265,942,016
Stats based on only the longest isoform per gene (unigenes)	
Contig N50	2258
Median contig length	444
Average contig	1045.24
Total assembled bases	64,290,538

**Table 4:** Statistics of simple sequence repeats (SSRs) identified by MISA.

Features	Transcriptome
Total number of sequences appraised	161,058
Total size of the sequences appraised (bp)	265,942,016
Total number of SSRs identified	27,066
Number of SSR-containing sequences	21,443
Number of sequences containing more than 1 SSR	4289
Number of SSRs in compound formation	2054
SSR frequency	1 SSR/9.825 kb

## Data Availability

The quantitative data and graphics (pictures) used to support the findings of this study are available.

## Additional Points

The raw sequence data has been deposited at the NCBI Short Read Archive (SRA) with accession numbers (SRR10120601, SRR10120602, SRR10120603, SRR10120604, SRR10120605, SRR10120606, SRR10120607, SRR10120608, SRR10120609, SRR10120610, SRR10120611, and SRR10120612).

## Supplementary Materials

Supplementary Materials Supporting figures: Figure S1A: data distribution. Supporting figures: Figure S1B: *E*-value distribution. Supporting figures: Figure S1C: number of sequences with length. Supporting figures: Figure S2A: the InterProScan ID distribution by the BlastProDom database. Supporting figures: Figure S2B: the InterProScan ID distribution by the FPrintScan database. Supporting figures: Figure S2C: the InterProScan ID distribution by the Smart database. Supporting figures: Figure S2D: the InterProScan ID distribution by the HAMAP database. Supporting figures: Figure S2E: the InterProScan ID distribution by the SuperFamily database. Supporting figures: Figure S2F: the InterProScan ID distribution by the Pfam database. Supporting figures: Figure S2G: the InterProScan ID distribution by the PANTHER database. Supporting figures: Figure S3A: enzyme code distribution of oxidoreductases. Supporting figures: Figure S3B: enzyme code distribution of transferases. Supporting figures: Figure S3C: enzyme code distribution of hydrolases. Supporting figures: Figure S3D: enzyme code distribution of lyases. Supporting figures: Figure S3E: enzyme code distribution of isomerases. Supporting figures: Figure S3F: enzyme code distribution of ligases.
