# Supplementary material for: Comprehensive Stress-Based De Novo Transcriptome Assembly and Annotation of Guar (Cyamopsis tetragonoloba (L.) Taub.): An Important Industrial and Forage Crop
Source: Int J Genomics. 2019 Oct 8;2019:7295859. doi: 10.1155/2019/7295859 (PMC6800914; doi:10.1155/2019/7295859)
Supplement: Supplementary Materials — Supporting figures: Figure S1A: data distribution. Supporting figures: Figure S1B: E-value distribution. Supporting figures: Figure S1C: number of sequences with length. Supporting figures: Figure S2A: the InterProScan ID distribution by the BlastProDom database. Supporting figures: Figure S2B: the InterProScan ID distribution by the FPrintScan database. Supporting figures: Figure S2C: the InterProScan ID distribution by the Smart database. Supporting figures: Figure S2D: the InterProScan ID distribution by the HAMAP database. Supporting figures: Figure S2E: the InterProScan ID distribution by the SuperFamily database. Supporting figures: Figure S2F: the InterProScan ID distribution by the Pfam database. Supporting figures: Figure S2G: the InterProScan ID distribution by the PANTHER database. Supporting figures: Figure S3A: enzyme code distribution of oxidoreductases. Supporting figures: Figure S3B: enzyme code distribution of transferases. Supporting figures: Figure S3C: enzyme code distribution of hydrolases. Supporting figures: Figure S3D: enzyme code distribution of lyases. Supporting figures: Figure S3E: enzyme code distribution of isomerases. Supporting figures: Figure S3F: enzyme code distribution of ligases. [file 7295859.f1.docx]

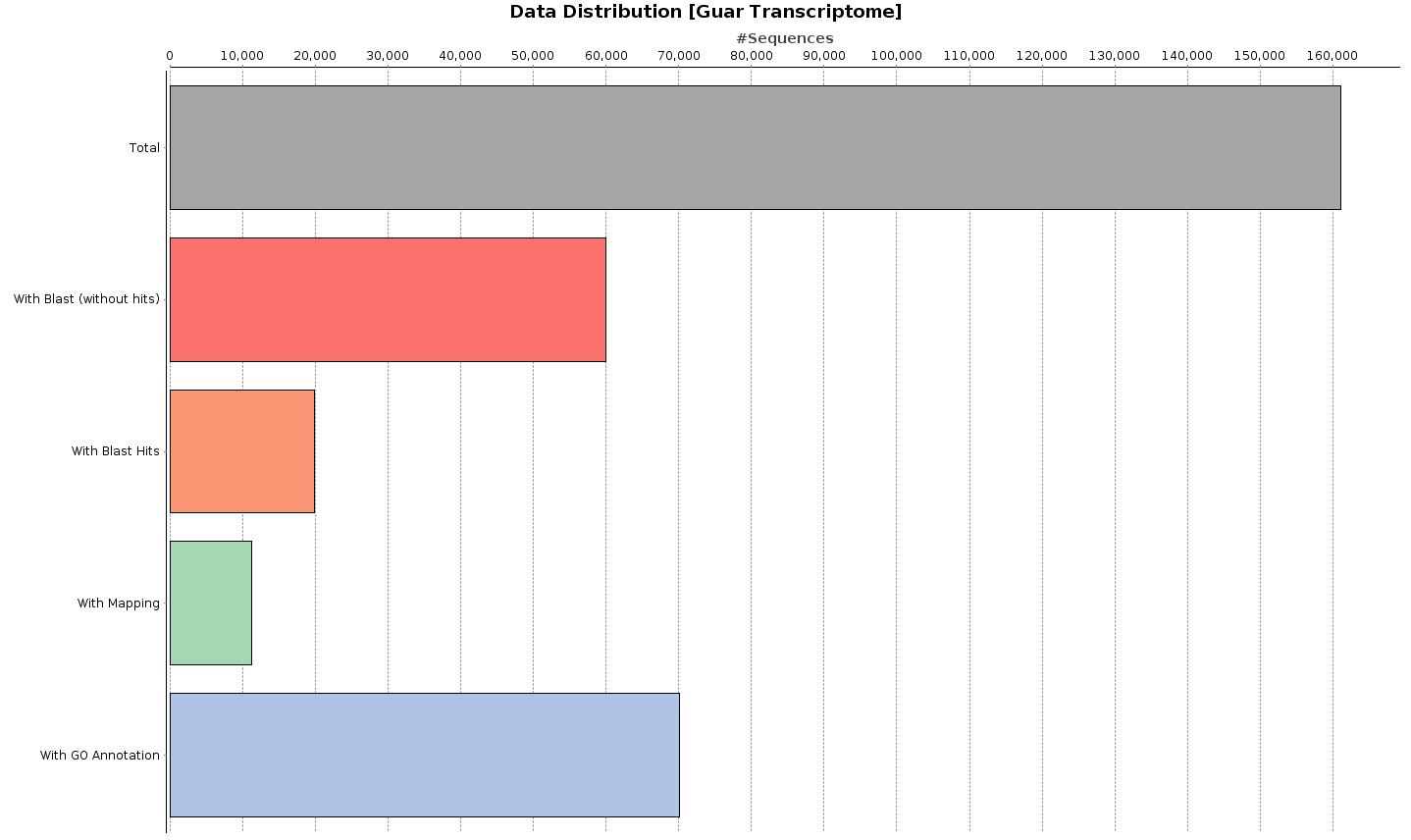


Figure S1A: Data Distribution


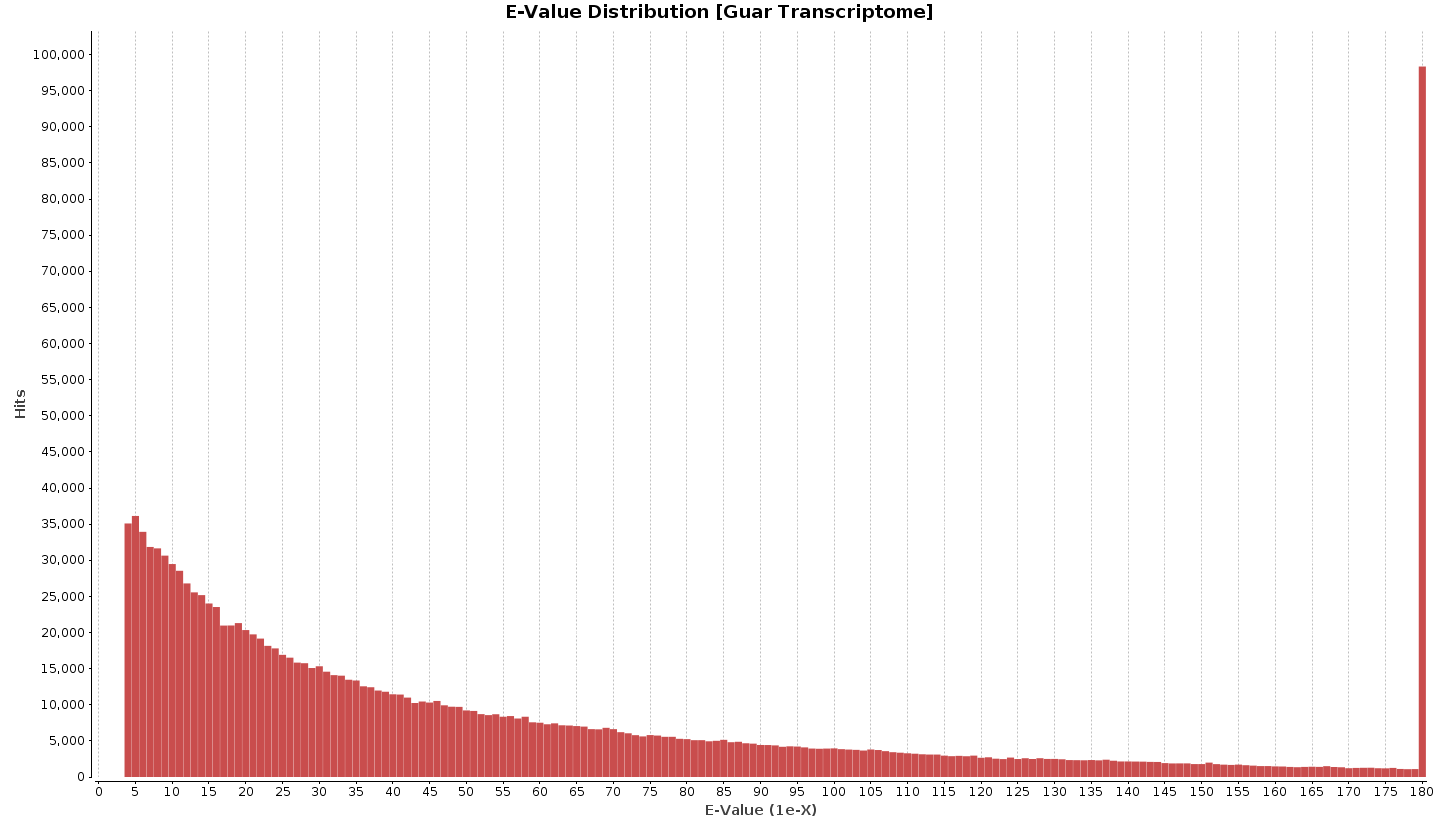


Figure S1B: E-value Distribution


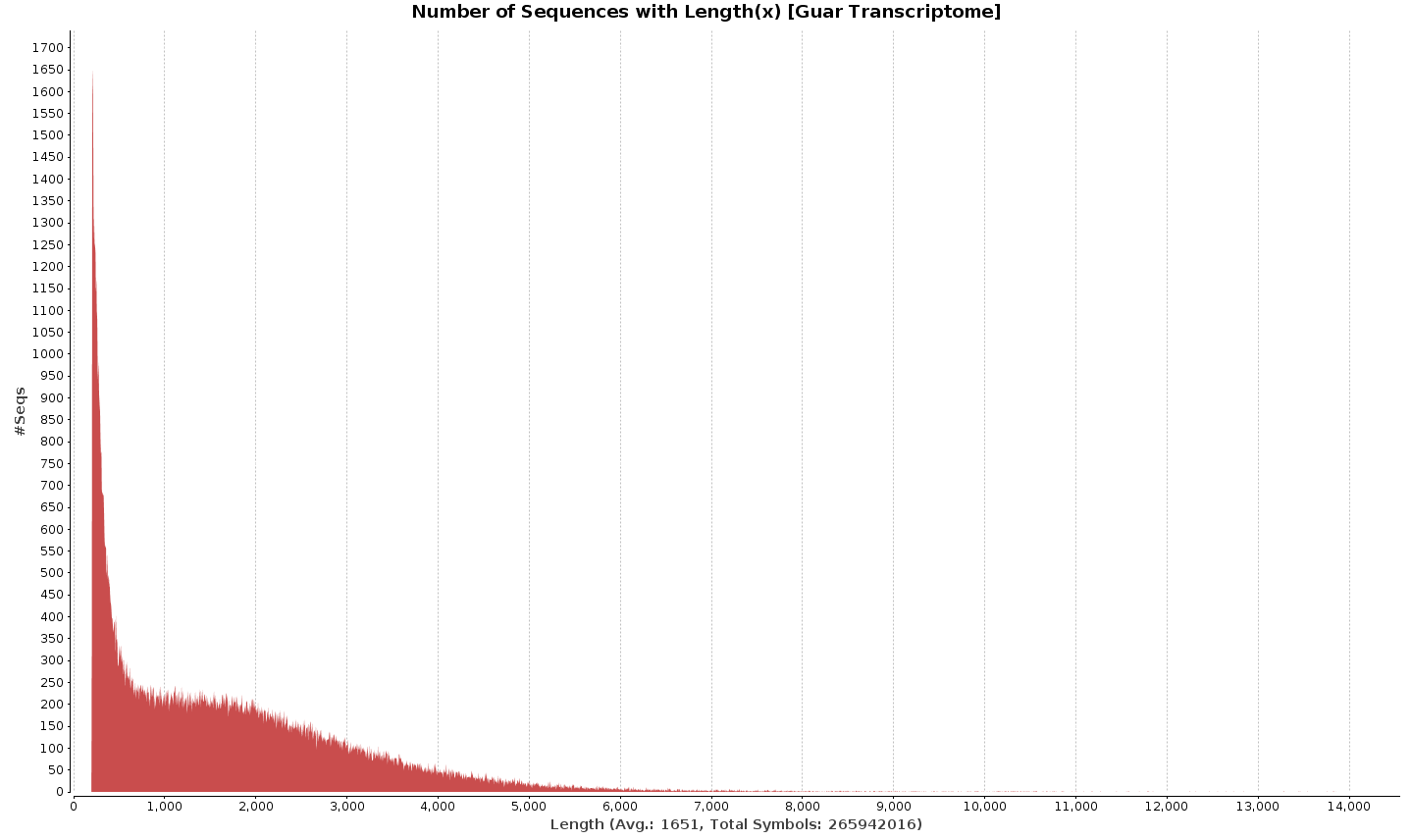


Figure S1C: Number of sequences with length


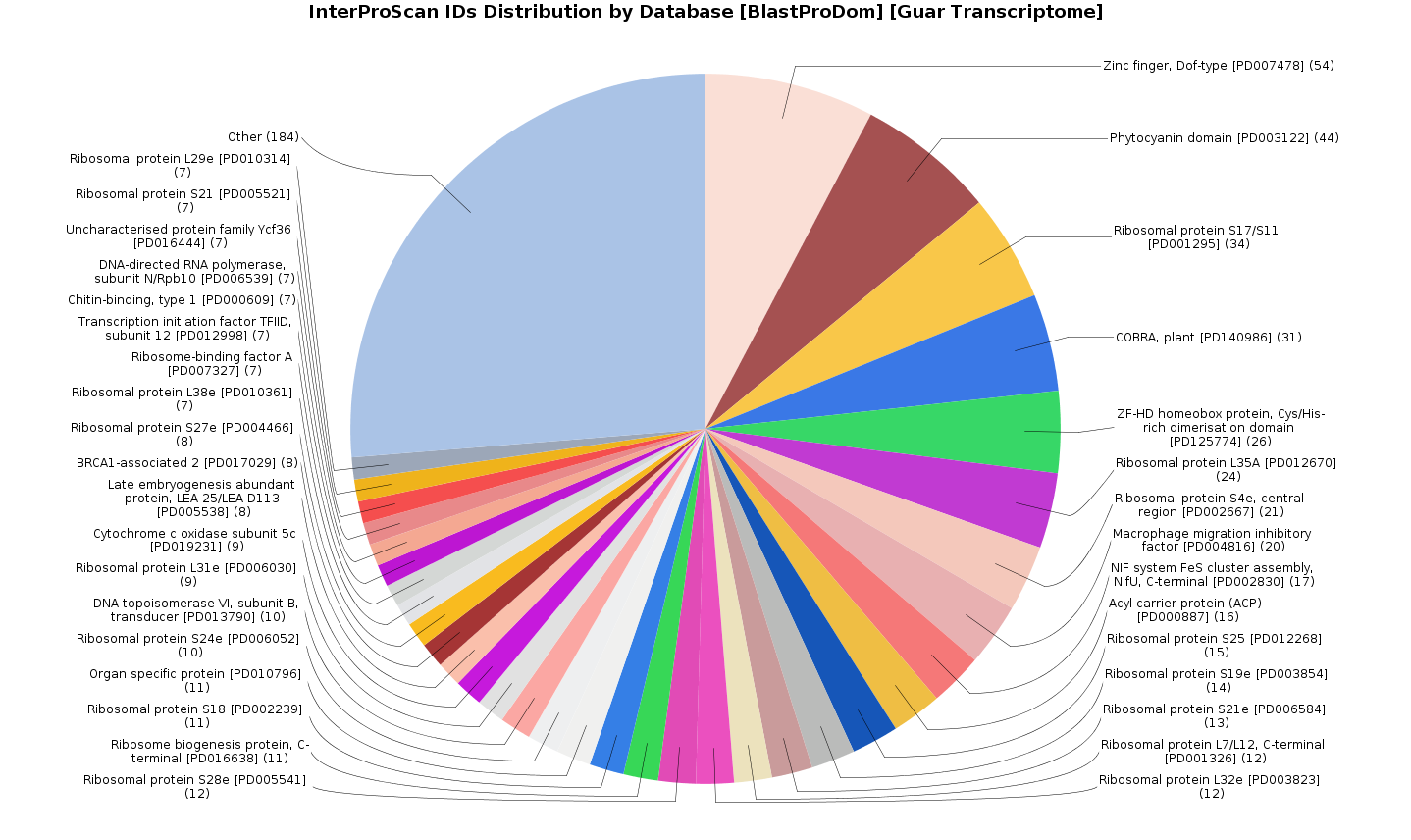


Figure S2A: The InterProScan IDs distribution by BlastProDom database


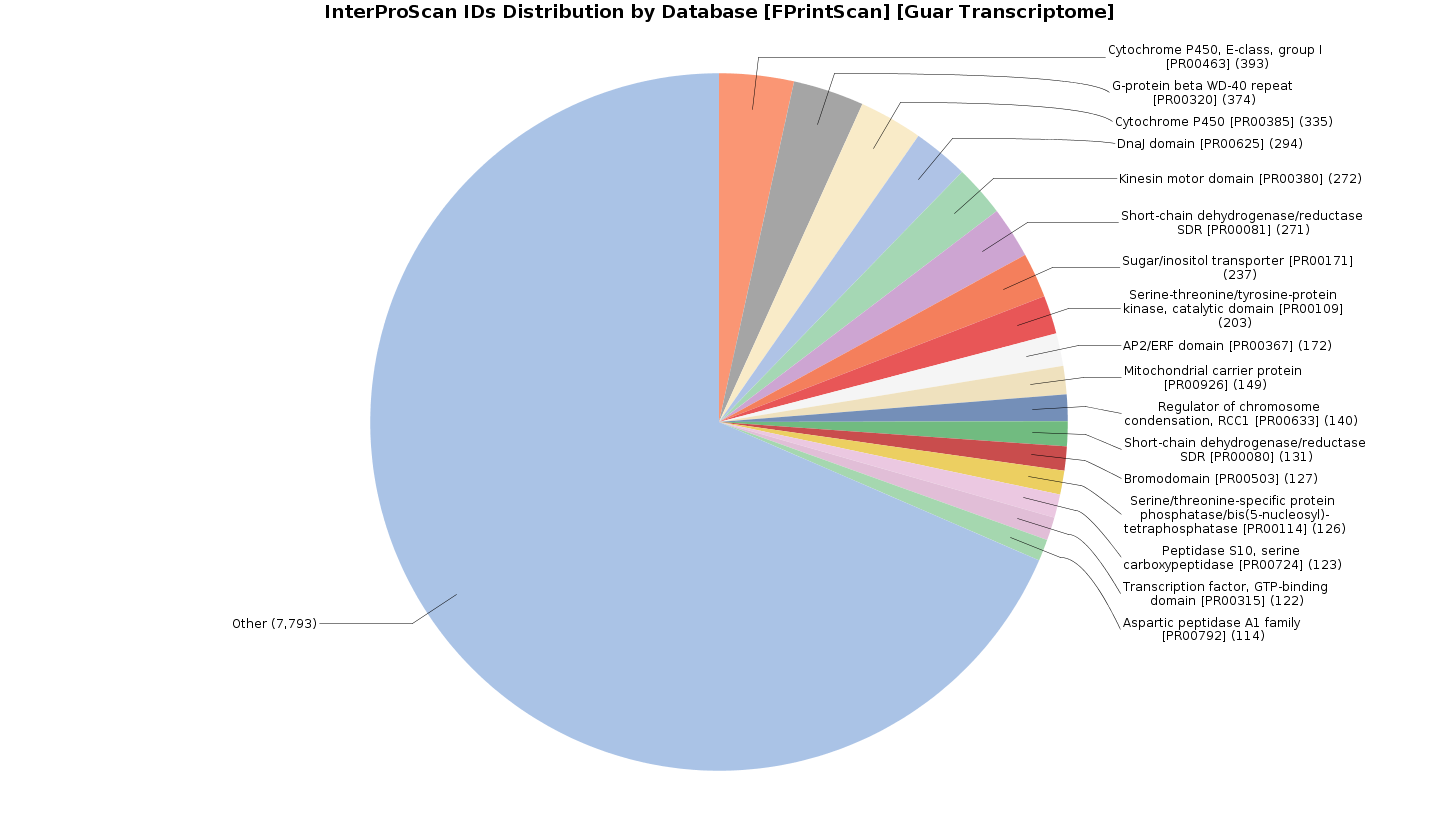


Figure S2B: The InterProScan IDs distribution by FPrintScan database


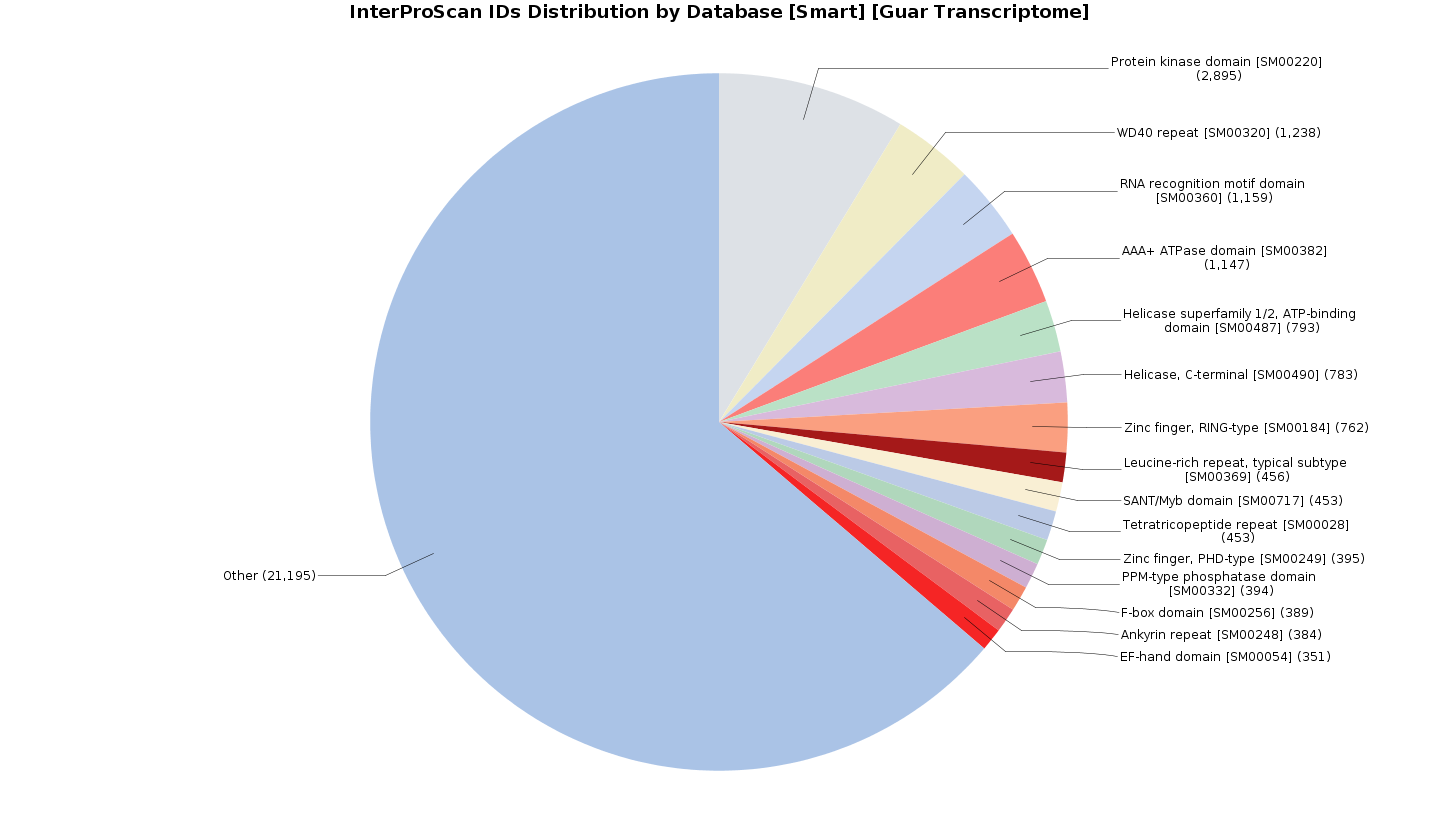


Figure S2C: The InterProScan IDs distribution by Smart database.


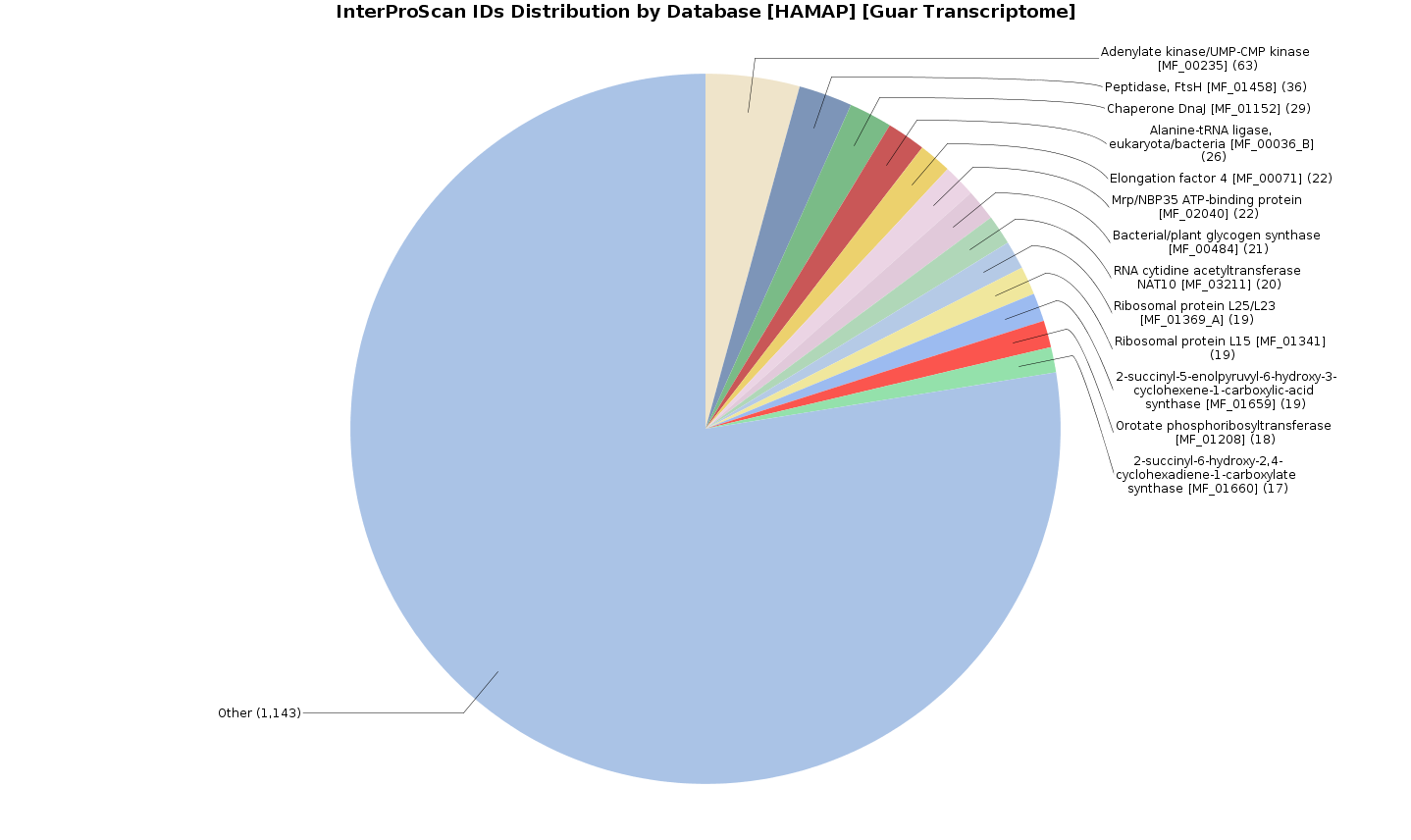


Figure S2D: The InterProScan IDs distribution by HAMAP database


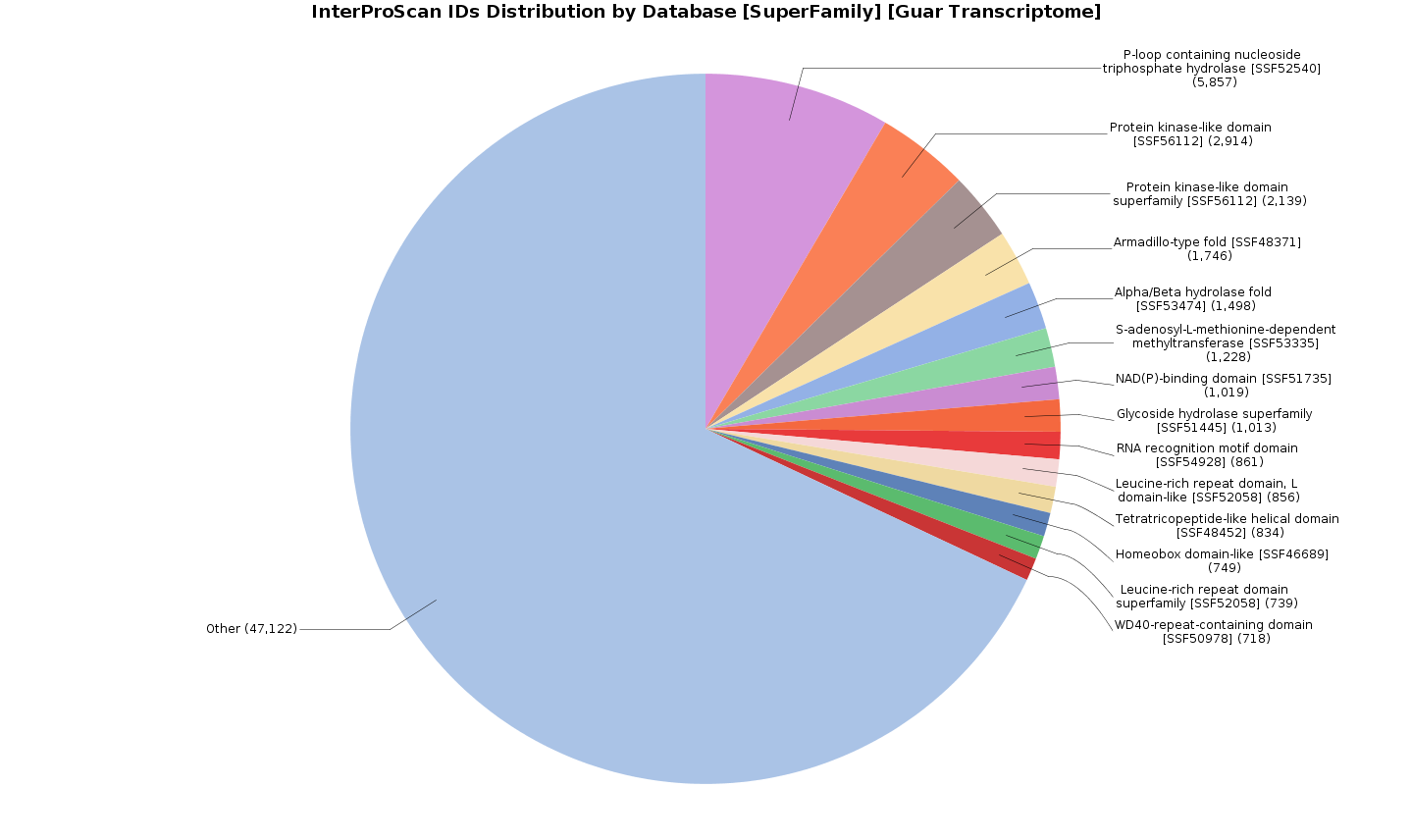


Figure S2E: The InterProScan IDs distribution by SuperFamily database.


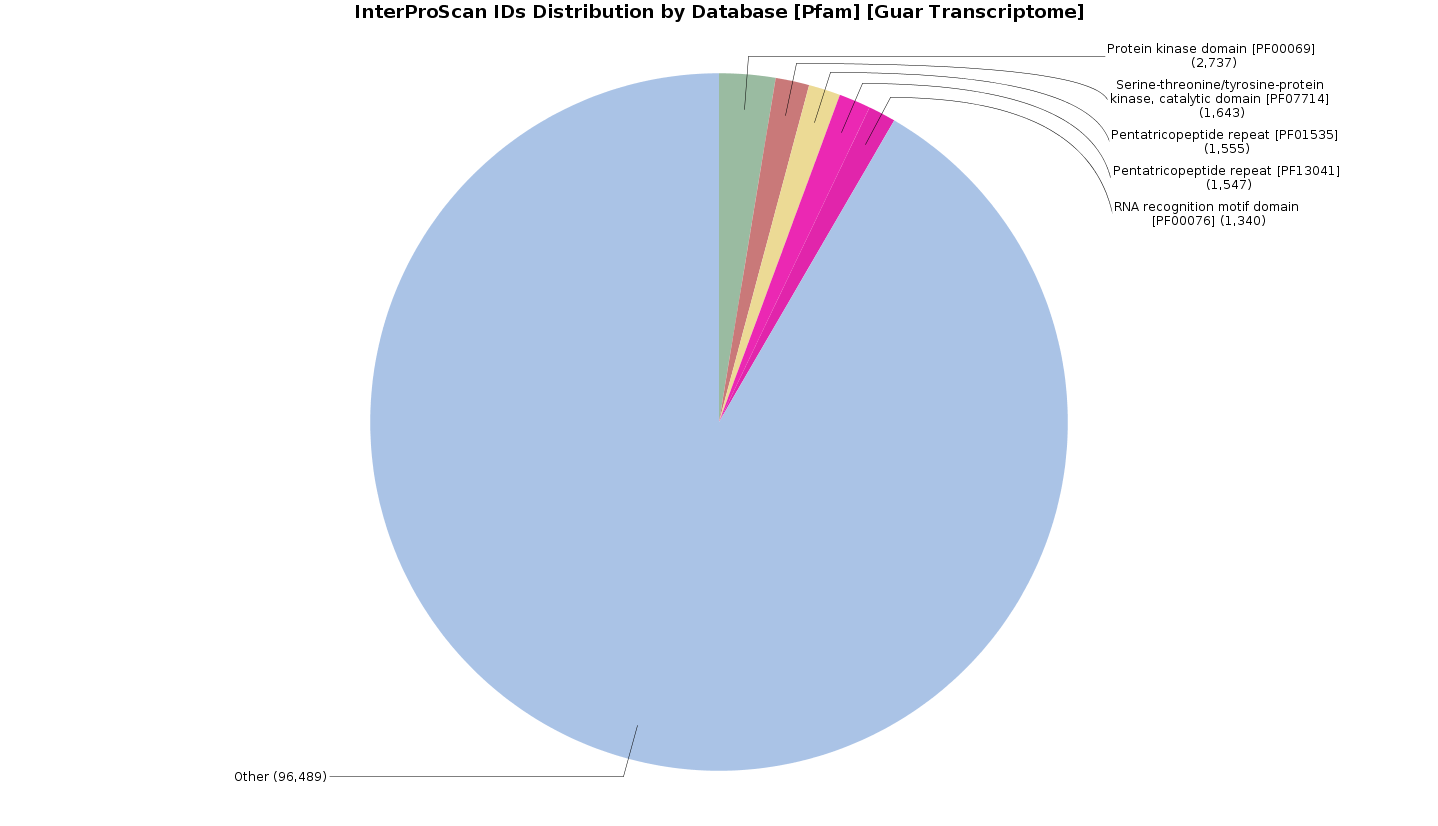


Figure S2F: The InterProScan IDs distribution by Pfam database.


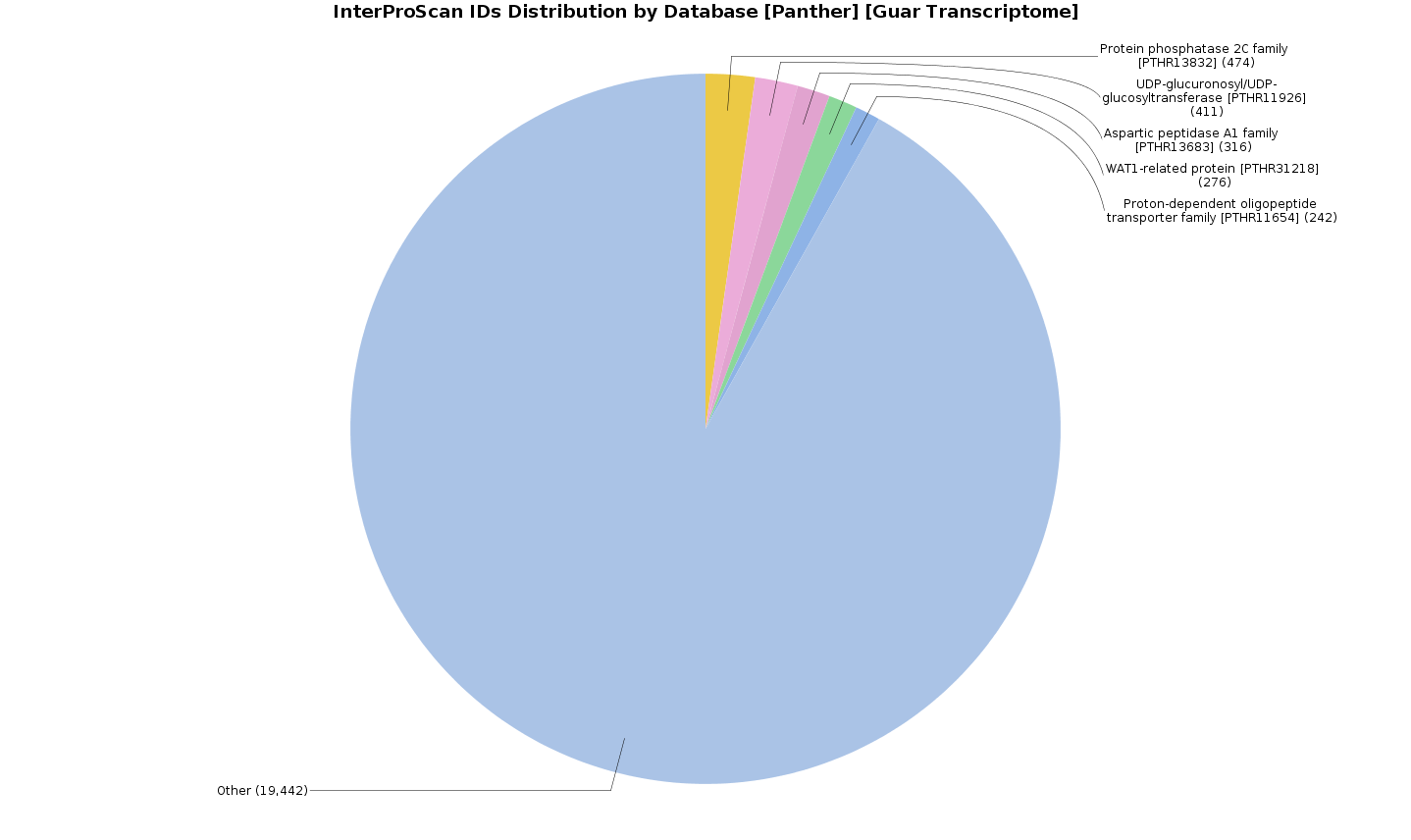


Figure S2G: The InterProScan IDs distribution by Panther database.


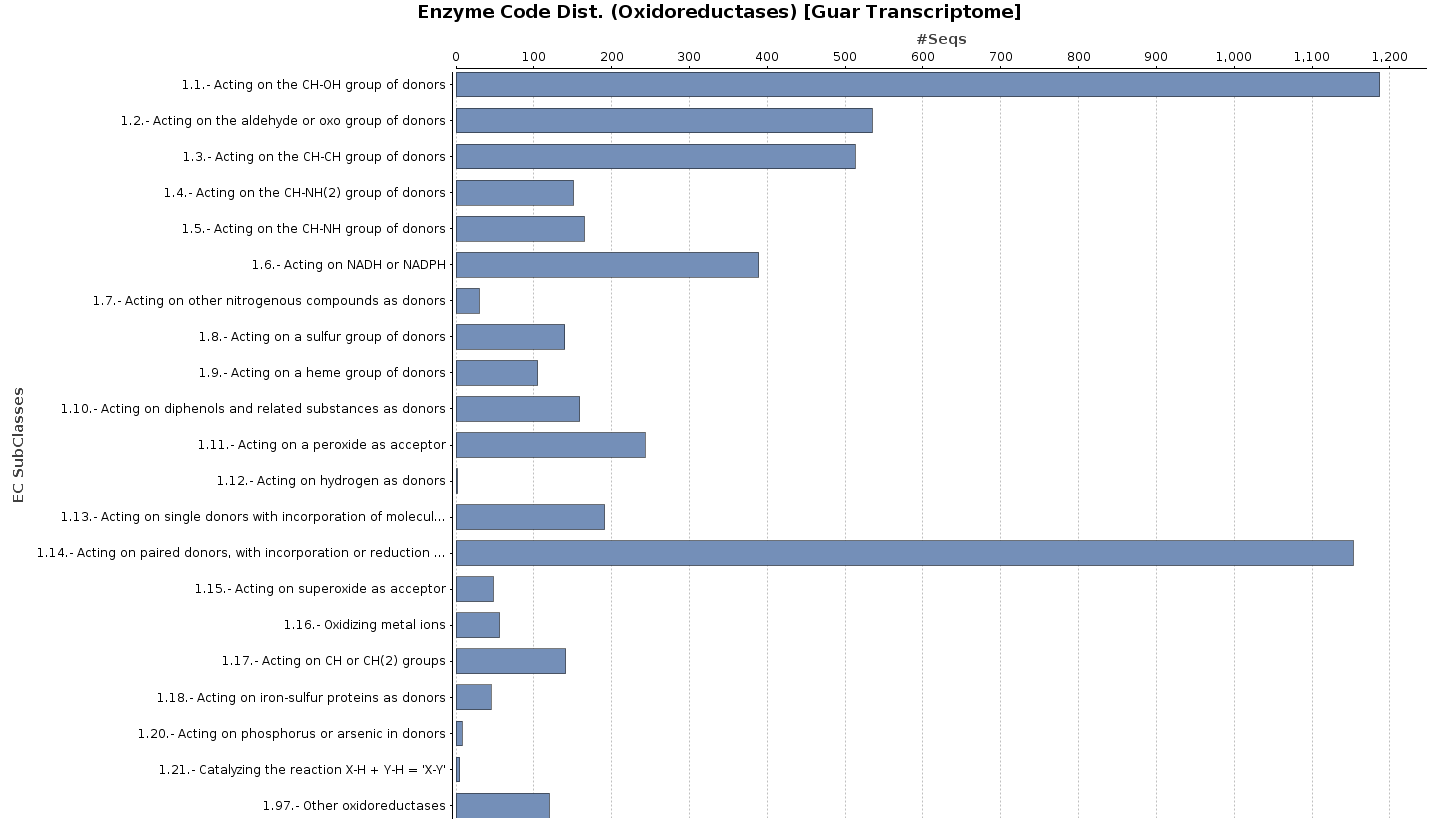


Figure S3A: Enzyme code distribution of oxidoreductases


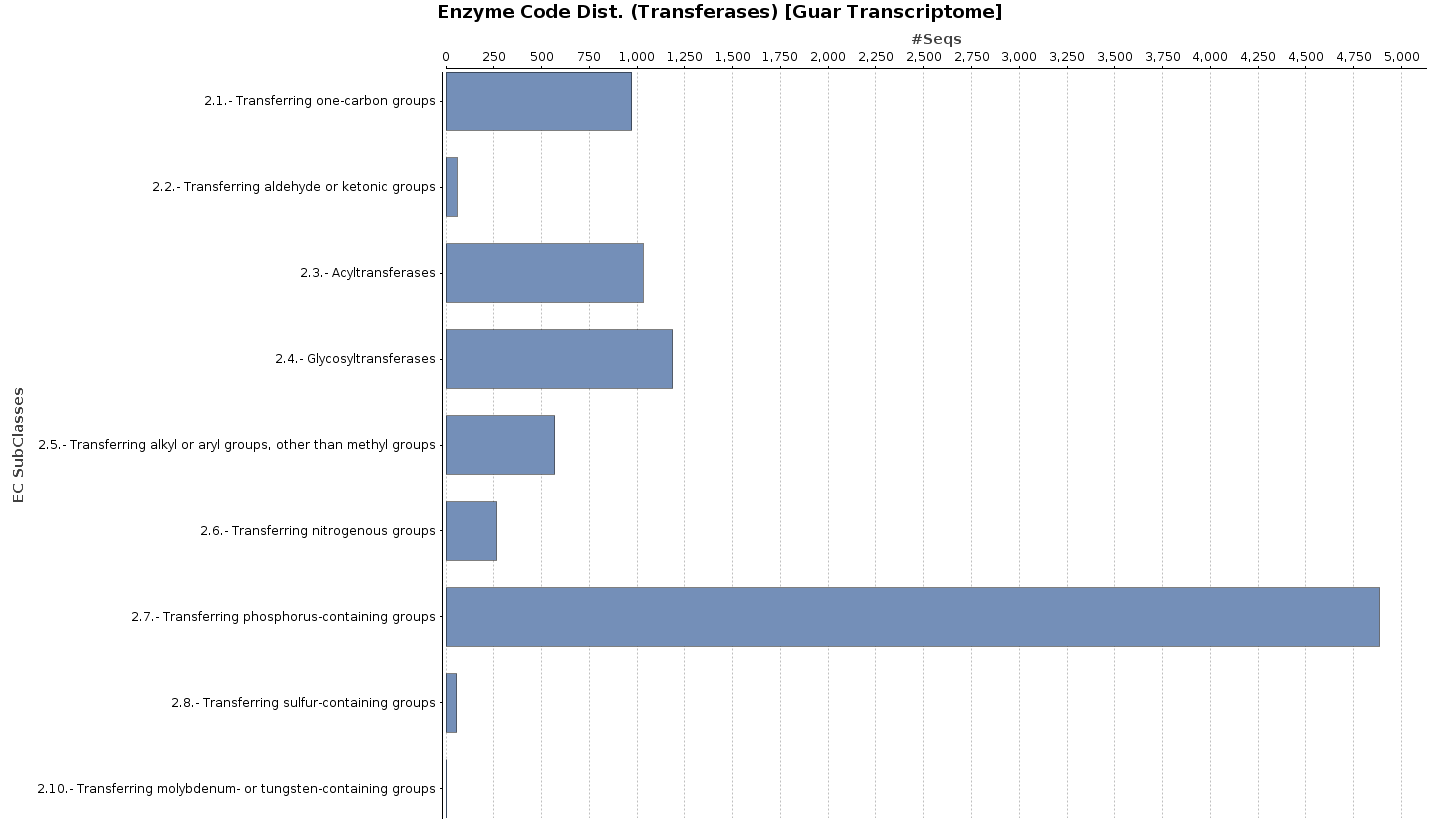


Figure S3B: Enzyme code distribution of transferases


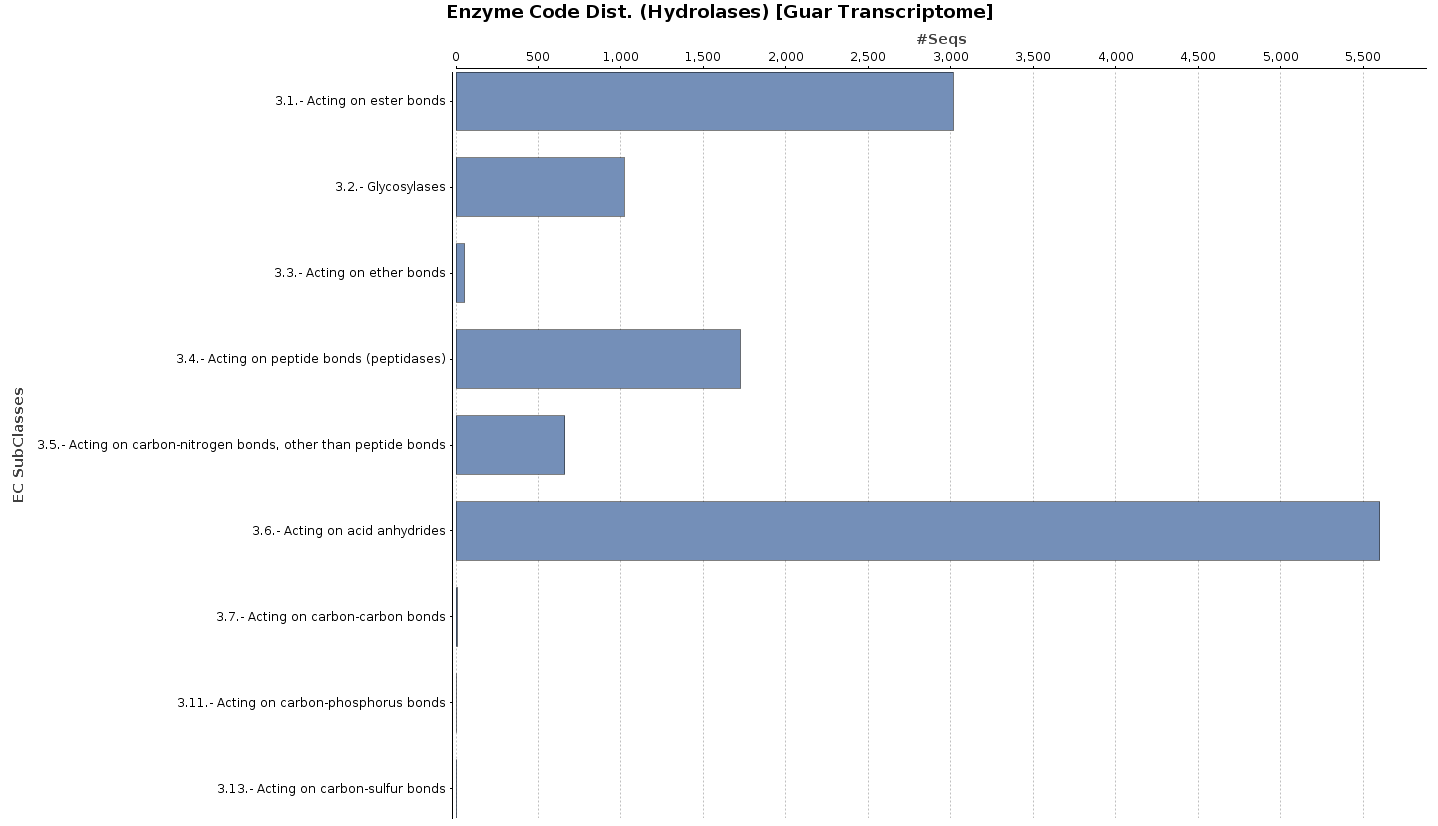


Figure S3C: Enzyme code distribution hydrolases.


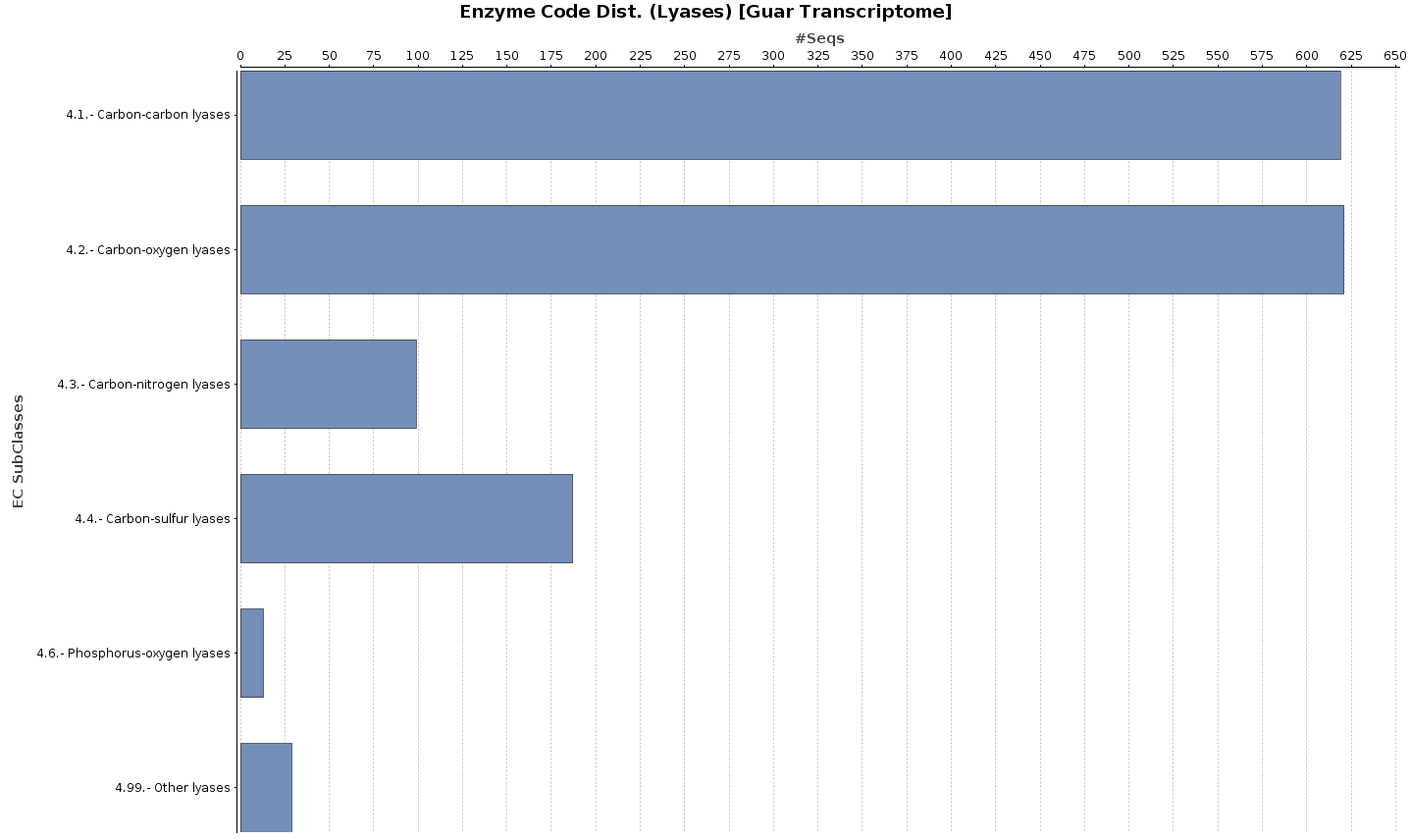


Figure S3D: Enzyme code distribution lyases


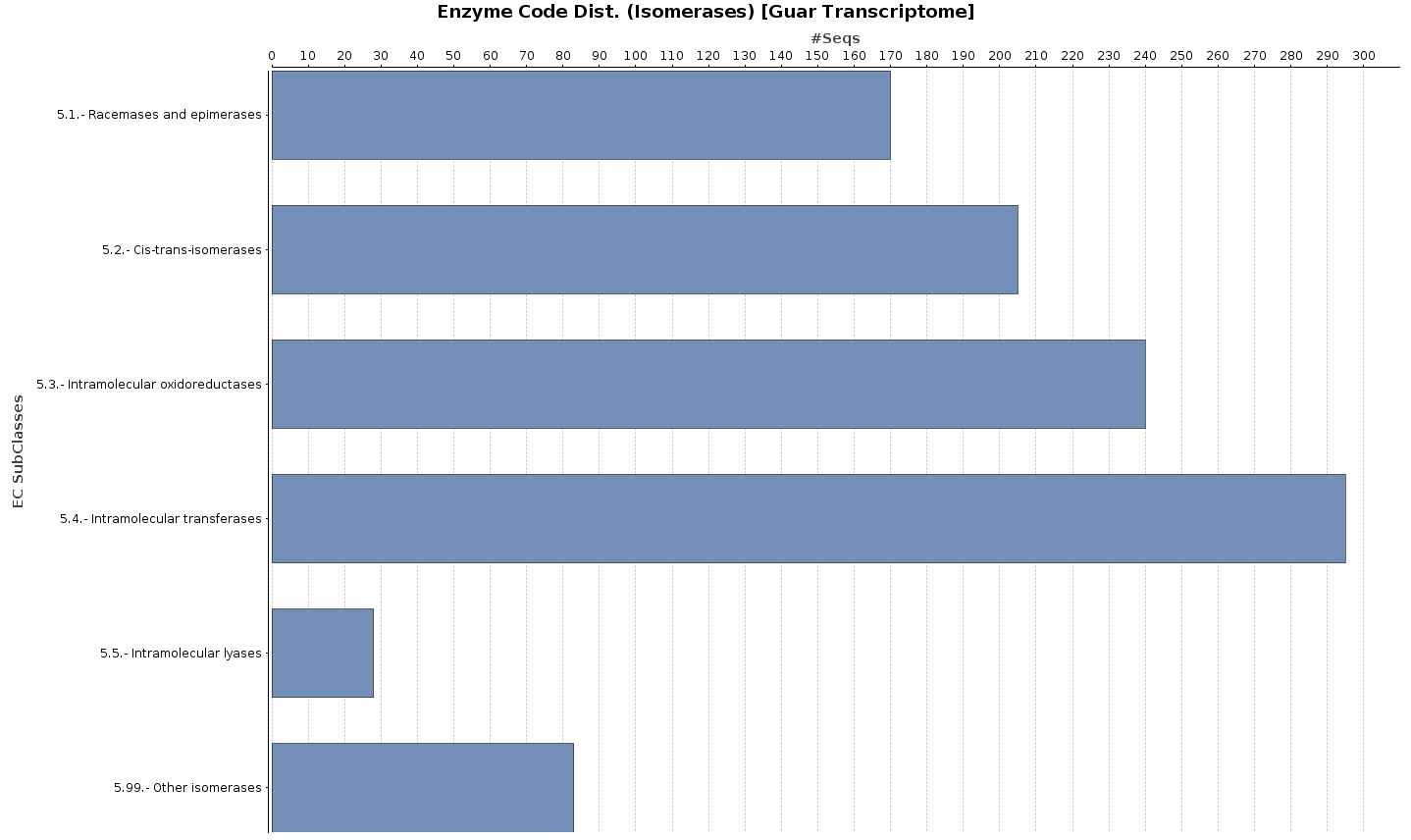


Figure S3E: Enzyme code distribution isomerases.


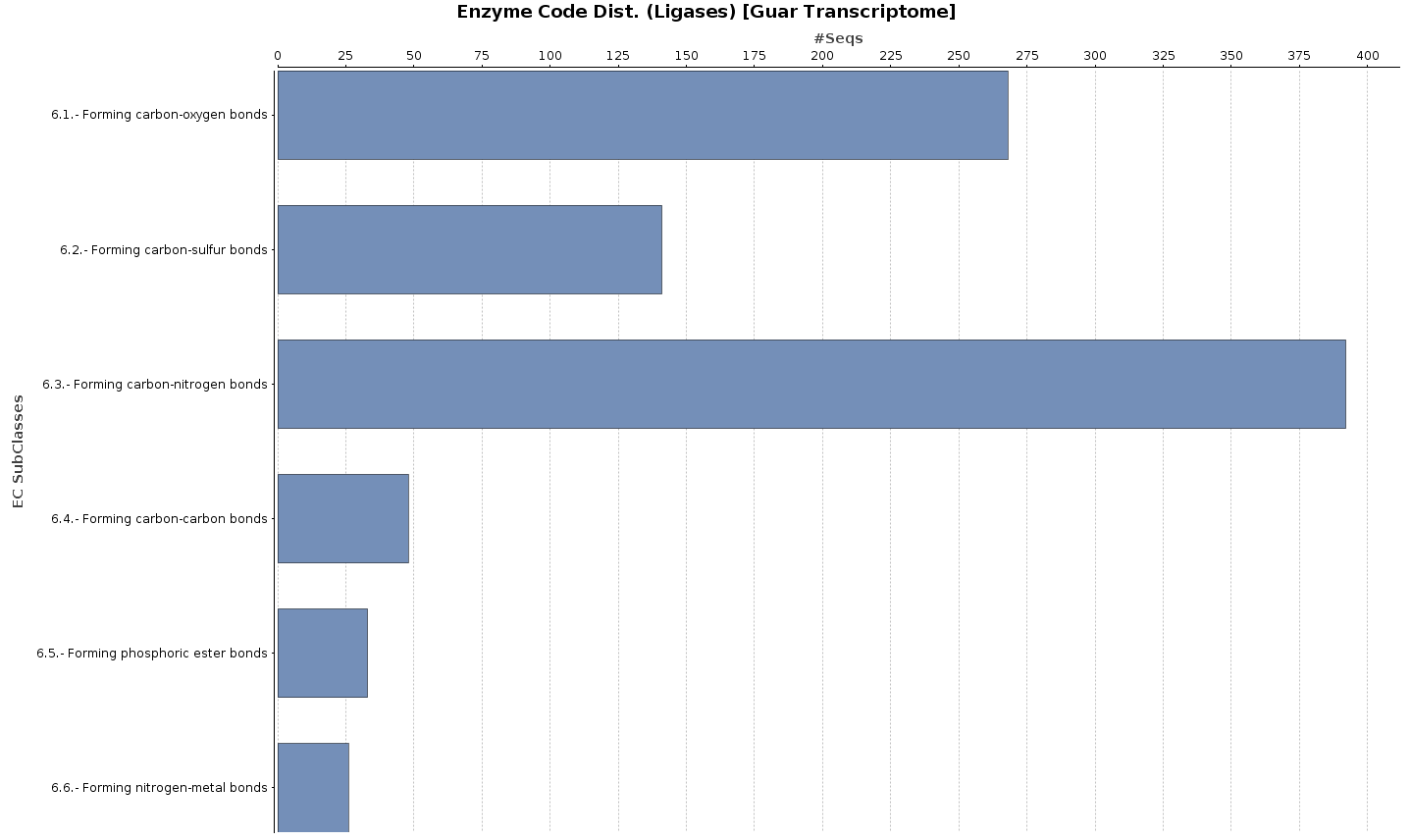


Figure S3F: Enzyme code distribution ligases.
